# Surprisingly frequent chromosomal instability in cultivated peanut

**DOI:** 10.1111/tpj.70617

**Published:** 2025-12-24

**Authors:** Samuele Lamon, Brian L. Abernathy, Soraya C. M. Leal‐Bertioli, David J. Bertioli

**Affiliations:** ^1^ Institute of Plant Breeding, Genetics and Genomics University of Georgia Athens Georgia 30602 USA; ^2^ Center for Applied Genetic Technologies University of Georgia Athens Georgia 30602 USA; ^3^ Department of Plant Pathology University of Georgia Athens Georgia 30602 USA; ^4^ Department of Crop & Soil Sciences University of Georgia Athens Georgia 30602 USA

**Keywords:** *Arachis hypogaea*, chromosomal instability, deletion, genome evolution, homeologous exchange, polyploidy, residual variation, spontaneous mutation, structural variation

## Abstract

This study, the third in a three‐part series, investigates whether chromosomal instability persists in cultivated peanut. The allotetraploid peanut (*Arachis hypogaea*; genome type AABB) originated from the hybridization and polyploidization of *A. duranensis* (AA) and *A. ipaënsis* (BB). Our first study established that this was an extremely narrow genetic origin, likely from a single hybridization event. This raised a paradox: how did such narrow genetics give rise to the phenotypic diversity seen in cultivated peanut? The second study addressed this, showing that a single neoallotetraploid spontaneously generates striking diversity, and that homoeologous exchanges—abundant in early generations following polyploidy—are a key mechanism in creating this diversity. In contrast to this early‐generation instability, cultivated peanut is generally considered to be genetically stable, presumably due to selection. This third study tests whether residual instability still occurs in modern peanut. From a single plant of the highly selfed ‘genome stock’ of the cultivar ‘Tifrunner’, we advanced lineages through seven generations in a pollinator‐free greenhouse. Among 233 plants, we identified three new large‐scale chromosomal instability events: a large deletion on chromosome B01, associated with reduced pod width and seed weight, and two ABBB compositions involving chromosomes A02/B02 and A05/B05. With these observations in hand, we reinterpreted previously published data from two recombinant inbred populations. Together, these results indicate that at least 1% of pure pedigree *A. hypogaea* plants exhibit spontaneous large‐scale chromosomal changes—a surprising frequency of instability that likely contributes to peanut's long‐term adaptability and evolution.

## Significance Statement

Although cultivated peanut is generally considered genetically stable, we show that spontaneous large‐scale chromosomal instability occurs in over 1% of individuals, even in pure pedigree lineages. These include unbalanced chromosome complements, supernumerary chromosomes, and deletions. One deletion observed in this study reduced pod and seed size, demonstrating phenotypic impact. This surprising level of residual genetic instability suggests that frequent structural variation shapes peanut evolution, with implications for breeding, genome interpretation, and its genome dynamics.

## INTRODUCTION

Peanut, also known as groundnut (*Arachis hypogaea* L.; AABB‐genome), presents a paradox. Although its origin was genetically very narrow, dating back less than 10 000 years ago, it has diversified into two subspecies, six botanical varieties, and thousands of landraces with differing growth habits, growing season lengths, pod morphologies, and seed sizes and colors (Krapovickas et al., 2009; Krapovickas & Gregory, 1994; Krapovickas & Gregory, 2007).

In this series of three manuscripts, the first reaffirmed peanut's extremely narrow origin (de Blas et al., 2025). The second resolved the paradox of peanut's morphological diversity by showing that a single neoallotetraploid hybrid—a close recreation of the original allotetraploid—spontaneously generated abundant genotypic and phenotypic variation and responded strongly to artificial selection (Lamon et al., 2025). In this third study, we examine to what degree traces of the genetic instability seen in neoallotetraploids remain detectable in a modern peanut cultivar (i.e., ‘Tifrunner’).

*Arachis hypogaea* is an allotetraploid (AABB, 2*n* = 4*x* = 40), with two almost complete chromosome sets derived from different diploid ancestors: the A‐subgenome from *A. duranensis* and the B‐subgenome from *A. ipaënsis*. These diploid species diverged relatively recently in evolutionary terms. The earliest estimate, based on limited sequence data, was 3.5 million years ago (Nielen et al., 2012). This was refined to 2.9 million years ago (Moretzsohn et al., 2013), and, with the availability of whole genome sequences, further narrowed to 2.2 million years ago—likely the most accurate time of divergence (Bertioli et al., 2016).

The A and B chromosome complements show striking similarities and notable differences. Nearly all chromosomes have simple one‐to‐one homeologous correspondence (Foncéka et al., 2009; Moretzsohn et al., 2009), and genic regions are approximately 98% identical. In contrast, intergenic regions have been extensively remodeled, largely through transposon activity. This creates a zipper‐like pattern of A–B synteny, with alternating regions of high identity and regions lacking direct correspondence (Bertioli et al., 2013). The divergence of the repetitive components of the A‐ and B‐genomes is highlighted by genomic *in situ* hybridization: labeled DNA probes from the ancestral diploids neatly distinguish the subgenomes of *A. hypogaea* (Seijo et al., 2007), an observation further supported by both case studies and broader surveys of repetitive elements (Nielen et al., 2010, 2012; Samoluk et al., 2015, 2019). In addition, the A‐genome lineage has undergone several large‐scale chromosomal inversions and, between two chromosomes, a complex reciprocal translocation (Bertioli et al., 2016; Ren et al., 2018). As foreseen by G.L. Stebbins (1947), when these chromosomes reside in the same nucleus, they display the hallmark features of a segmental allotetraploid: sufficient divergence to promote preferential homologous pairing, yet enough similarity—particularly in gene‐rich regions—to permit occasional homeologous interactions. Although interaction between the A and B genomes was implicit in Husted's cytogenetic work of the 1930s (1933, 1936)—where low but significant frequencies of non‐bivalent chromosome associations were observed—the potential for genetic exchange was long overlooked in modern peanut genetics. It was first demonstrated through detailed analysis of marker segregation and expression data by Leal‐Bertioli et al. (2015). Later, genome sequencing of *A. hypogaea* revealed its signatures: in some terminal chromosome regions, homeologous exchange has converted them to AAAA structure, and to a lesser extent, BBBB (Bertioli et al., 2016, 2019).

The close recreation of the initial tetraploid that gave rise to *A. hypogaea*, by hybridization of *A. ipaënsis* and *A. duranensis* followed by chromosome doubling (Fávero et al., 2006) opened new perspectives in peanut research. Genetic analysis revealed recombination between the A and B genomes following polyploidy (Leal‐Bertioli et al., 2015, 2021). This has now been shown to generate heritable variation through homeologous recombination—even from a genetically uniform initial hybrid. This finding, described in the second of this series of three papers, illustrates a tangible polyploid advantage for domestication (Lamon et al., 2025).

Despite a likely genetically tumultuous period following polyploidization, cultivated peanut is now generally considered morphologically and genetically stable—a pattern of stabilization over time that is observed in many polyploids (Wendel, 2000). However, some observations of recombination between A‐ and B‐subgenomes have been made (Bertioli et al., 2016). The purpose of this study was to investigate to what extent the genetic instability of neoallotetraploids persists in *A. hypogaea*.

To test this, from a single plant, we advanced lineages of the highly selfed ‘genome stock’ of the tetraploid reference genotype, *A. hypogaea* cv. ‘Tifrunner’ (Bertioli et al., 2019; Holbrook & Culbreath, 2007), through seven generations under carefully controlled conditions, with individual plant harvests and single‐seed descent. The entire experiment was conducted in a pollinator‐free greenhouse—to eliminate any confounding influence of troublesome bees—allowing us to track lineages with confidence. Our analysis revealed a surprising degree of large‐scale chromosomal instability in cultivated peanut, indicating that structural variation continues to contribute to heritable diversity. Thus, the resolution of peanut's morphological diversity paradox may continue to lie in the behavior of its polyploid genome, persisting long after its origin.

## RESULTS

### Spontaneous genome instabilities detected by genotyping

**Figure 1:**
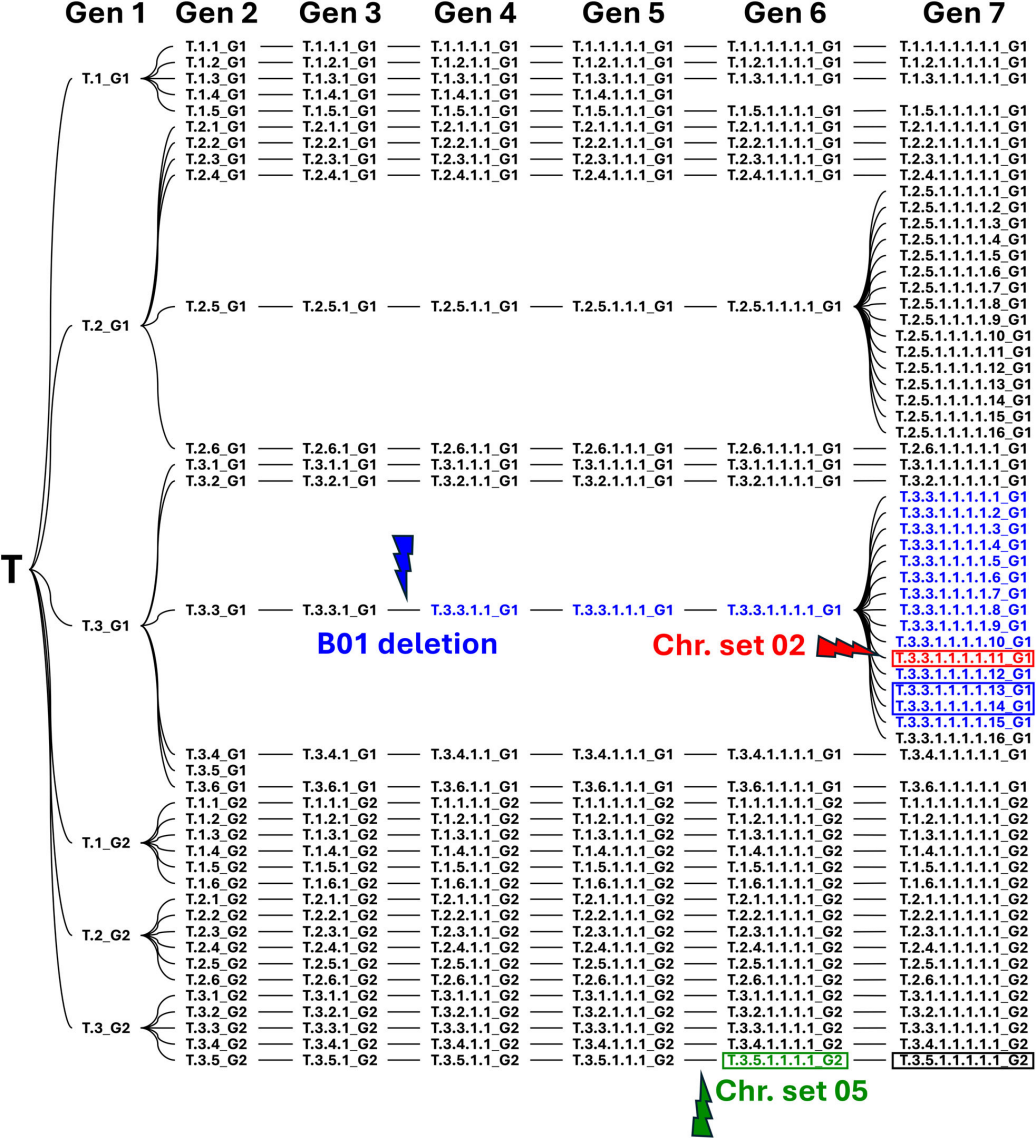
Pedigree of *Arachis hypogaea* cv. ‘Tifrunner’ lineages advanced through seven generations by single‐seed descent. All plants were derived from a single plant of the highly selfed ‘Tifrunner’ ‘genome stock.’ Each plant was assigned a unique identifier encoding its pedigree, with one digit added per generation. The suffix ‘_G1’ or ‘_G2’ indicates the greenhouse in which the plant was grown. Colors indicate plants with spontaneous structural variants: blue shows plants with a deletion at the top of chromosome B01 (first detected in generation 4); red marks a plant that carries the B01 deletion and also an ABBB composition along chromosome set 02; green marks a plant with a transient ABBB composition on chromosome set 05. Black indicates plants without detected large‐scale structural changes. Plants selected for whole genome sequencing are outlined in boxes.

Starting with seeds from a single plant of the highly selfed genome stock, *A. hypogaea* cv. ‘Tifrunner’ lineages were advanced over seven generations (Figure 1). To systematically track plants and lineages, a naming convention was implemented. For instance, the identifier T.3.3.1.1.1.1.11_G1 denotes ‘T’ as the original ‘Tifrunner’ plant, followed by a series of numbers representing single‐seed descent in seven generations. Lastly, the ‘_G1’ suffix indicates cultivation in greenhouse 1 and ‘_G2’, cultivation in greenhouse 2.

**Figure 2:**
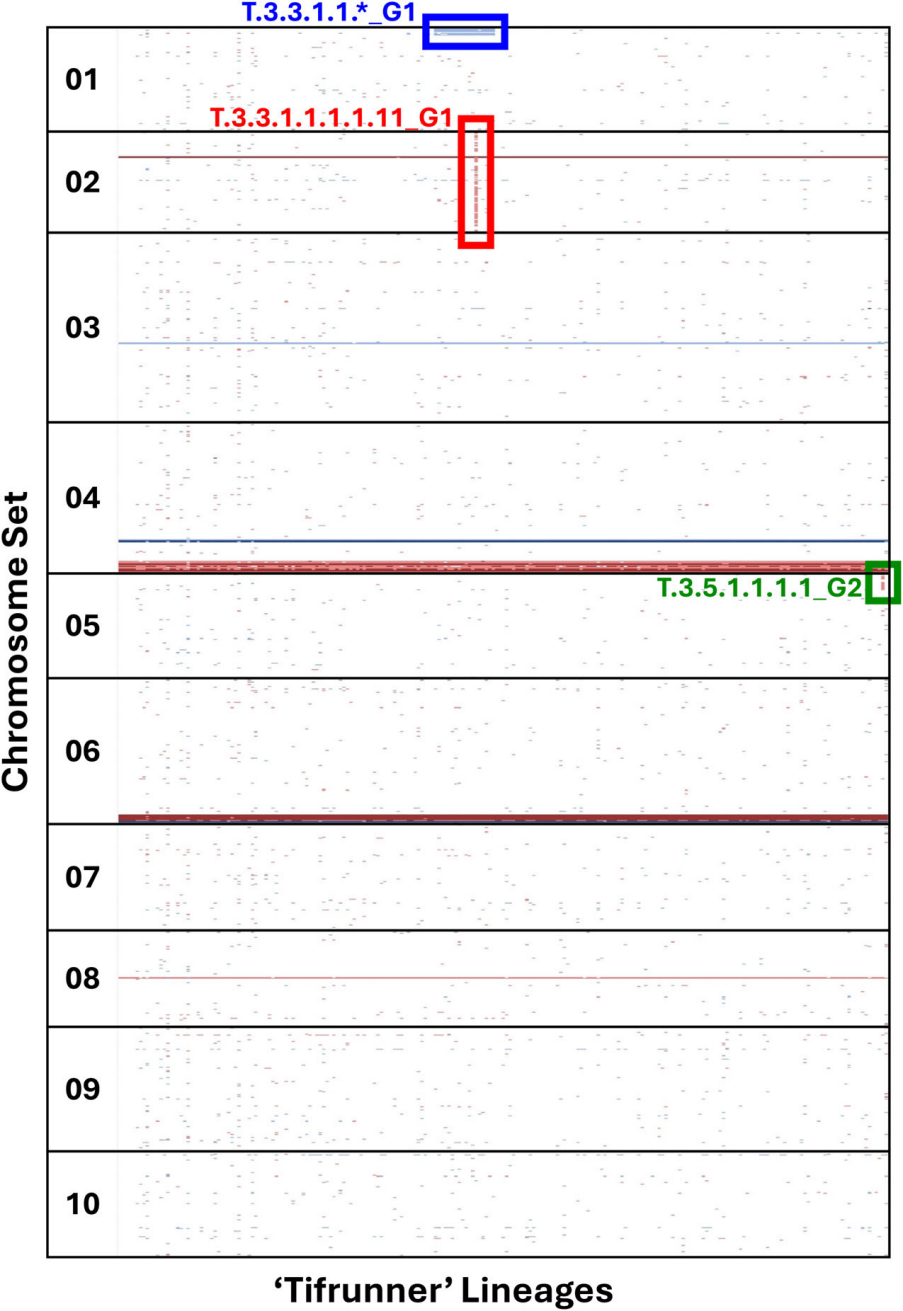
Genotyping reveals spontaneous structural variants in *Arachis hypogaea* cv. ‘Tifrunner’. Each column represents a ‘Tifrunner’ plant, and SNP markers are arranged in rows according to their positions on the *A. ipaënsis* gnm1 reference genome. The y‐axis groups markers by chromosome sets 1–10, each representing the combined signal from A‐ and B‐subgenome homeologs. Color indicates inferred genome dosage: BBBB in dark red, ABBB in light red, AAAB in light blue, AAAA in dark blue. The red box highlights an ABBB composition across chromosome set 02. The blue box marks a region at the top of chromosome set 01 miscalled as AAAB by SNP‐based genotyping, but later shown by sequencing to reflect a B01 deletion. The green box marks an ABBB composition on chromosome set 05.

Genotyping of the ‘Tifrunner’ plants was visualized using a color‐coded matrix (Figure 2). To facilitate visualization, markers were grouped by chromosome sets, numbered 1 to 10, each set corresponding to a pair of homeologous chromosomes (e.g., A01 and B01 form chromosome set 01). This approach highlights dosage changes across homeologous pairs. The typical genomic composition for most of the ‘Tifrunner’ genome is AABB, with each chromosome set containing two copies of the A‐subgenome and two copies of the B‐subgenome. This balanced AABB pattern is observed across most chromosomal regions. At some chromosome terminal regions, all ‘Tifrunner’ plants have specific unbalanced genome compositions previously described in the ‘Tifrunner’ reference genome (Bertioli et al., 2019). However, in some plants and in some chromosomes we detected novel unexpected genome compositions, which are highlighted in the color‐coded matrix as deviations from the standard AABB composition.

An abnormal composition was identified at the top of chromosome set 01 in T.3.3.1.1_G1 and its descendants, later revealed by sequencing to reflect a B01 deletion at the top of the chromosome. In another plant, T.3.3.1.1.1.1.11_G1, an ABBB pattern across all of chromosome set 02 was observed, in addition to the deletion in chromosome B01 inherited from T.3.3.1.1_G1. In another case, plant T.3.5.1.1.1.1_G2 displayed an ABBB composition on chromosome set 05. Across all generations, with these 3 events detected among 233 plants, the overall rate of novel large‐scale structural instability events was 1.3%, (Additional File 2: Table S1).

**Figure 3:**
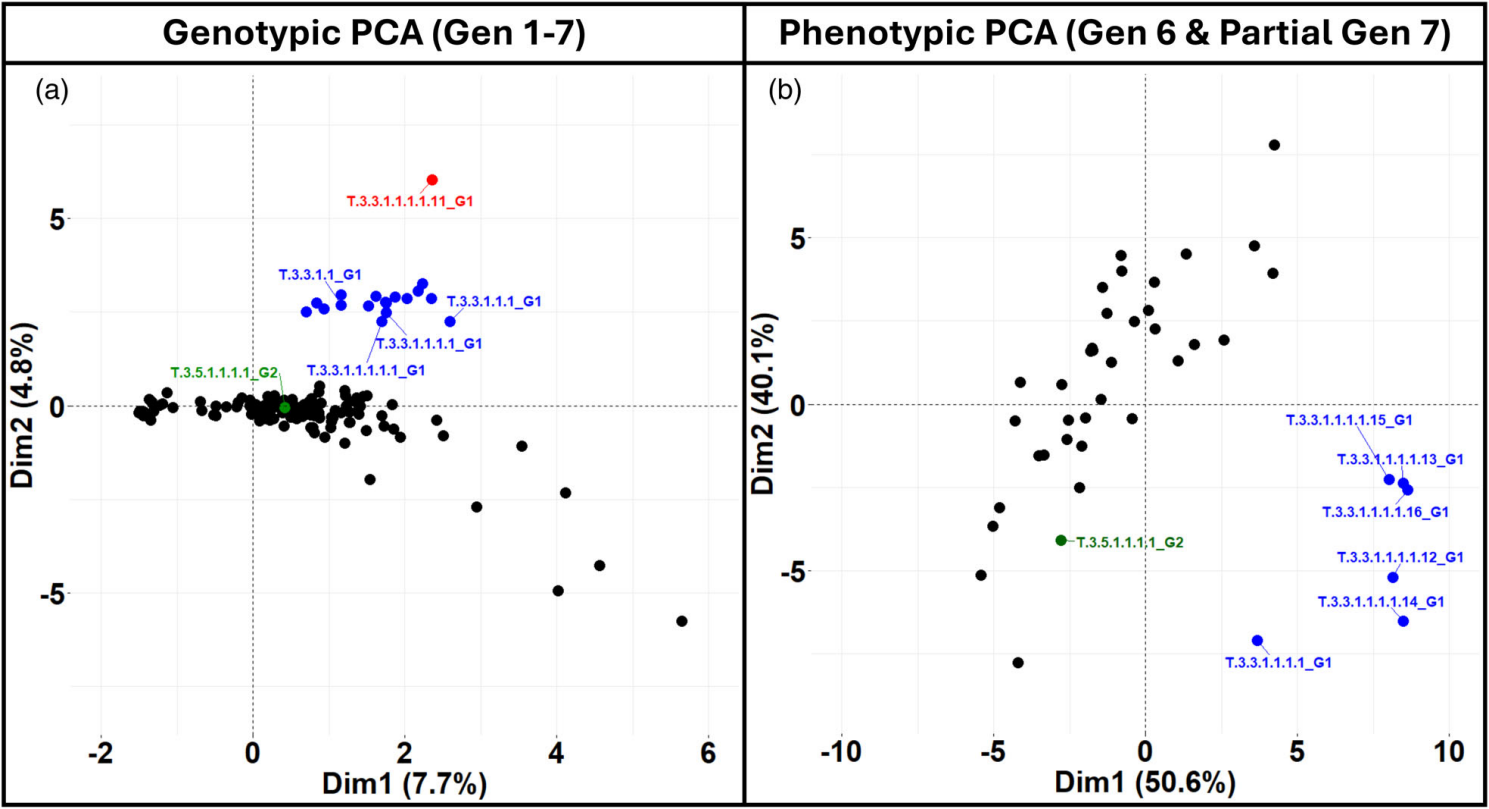
Spontaneous genetic and phenotypic variation arises in pure pedigree *Arachis hypogaea* cv ‘Tifrunner’. (a) Shows a principal component analysis (PCA) of genome‐wide genotype data from generations 1 to 7. (b) Shows PCA of pod morphology traits from generation 6 and a subset of generation 7. Black dots represent plants without novel large‐scale structural changes. Blue dots indicate plants carrying a deletion on chromosome B01, which arose spontaneously in generation 4. The red dot represents a plant from generation 7 that inherited the B01 deletion and also carries an ABBB composition along chromosome set 02. The green dot marks a plant with a transient ABBB composition on chromosome set 05, first observed in generation 6.

Principal component analysis separated the genotypes according to the structural changes (Figure 3a; Additional File 1: Figure S1).

### Analysis of the spontaneous genome instabilities by whole genome sequencing

**Figure 4:**
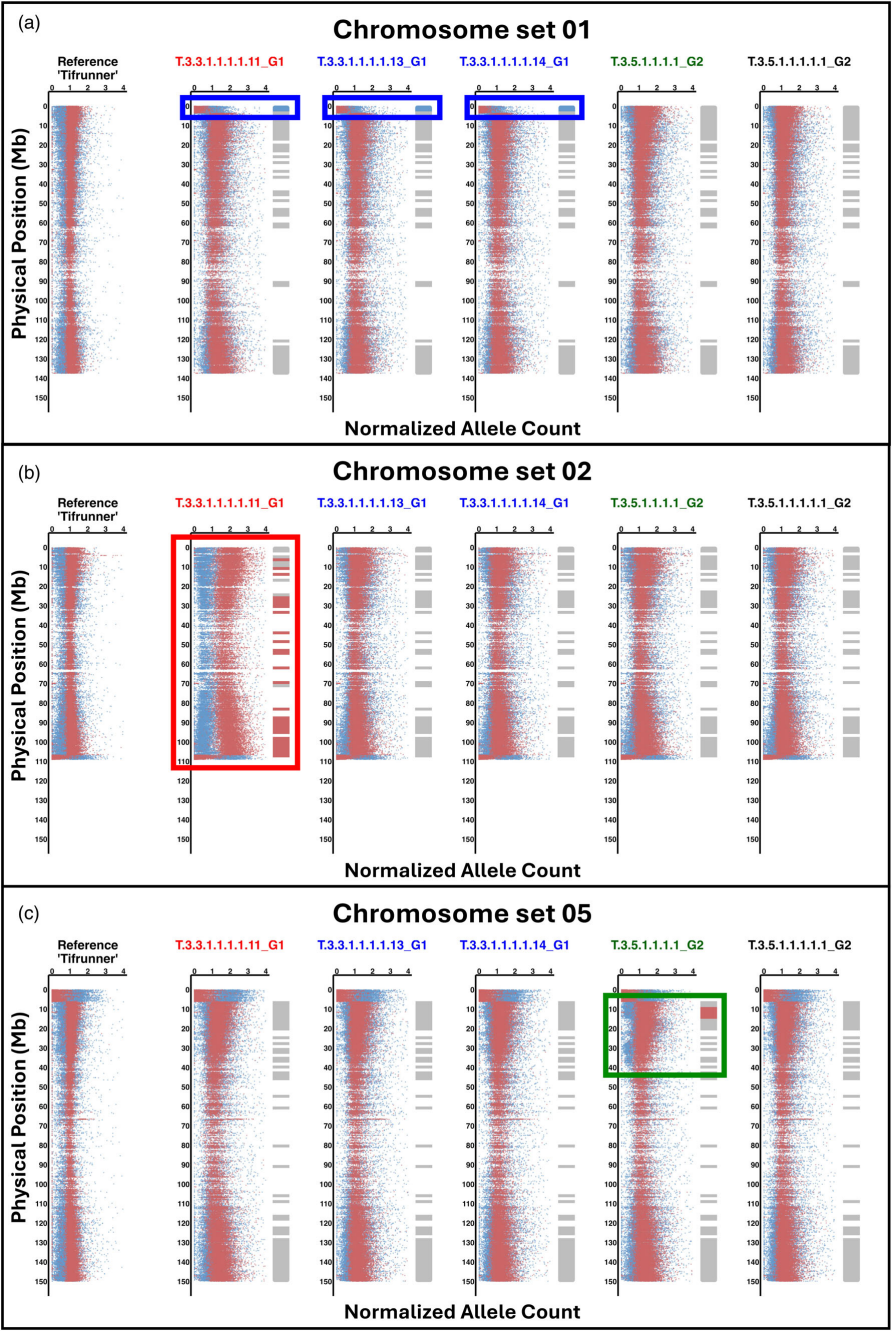
Sequencing confirms the structural variants detected by SNP‐based genotyping. (a–c) show normalized allele counts from whole genome sequencing (left side of each track) and SNP‐based genotyping results (bars, right side of each track) for chromosome sets 01 (a), 02 (b), and 05 (c) in the ‘Tifrunner’ reference and selected plants. In SNP‐based genotyping, genomic dosage imbalances are color‐coded: BBBB regions in dark red, ABBB in light red, AAAB in light blue, and AAAA in dark blue. Balanced genomic compositions (AABB) are shown in gray. In sequencing, A‐genome allele dosage is shown as blue dots; B‐genome dosage as red dots. Balanced regions (AABB) show equal representation of A and B alleles, while deviations indicate structural variants: deletions (e.g., B01, blue box) and abnormal chromosome compositions ABBB (e.g., chromosome set 02, red box; 05, green box). T.3.3.1.1.1.1.11_G1 carries both a deletion at the top of chromosome B01 and an ABBB composition across chromosome set 02. T.3.3.1.1.1.1.13_G1 and T.3.3.1.1.1.1.14_G1 also carry the B01 deletion. An ABBB composition on chromosome set 05 was observed in T.3.5.1.1.1.1_G2 (green box) but not in its progeny, T.3.5.1.1.1.1.1_G2.

**Figure 5:**
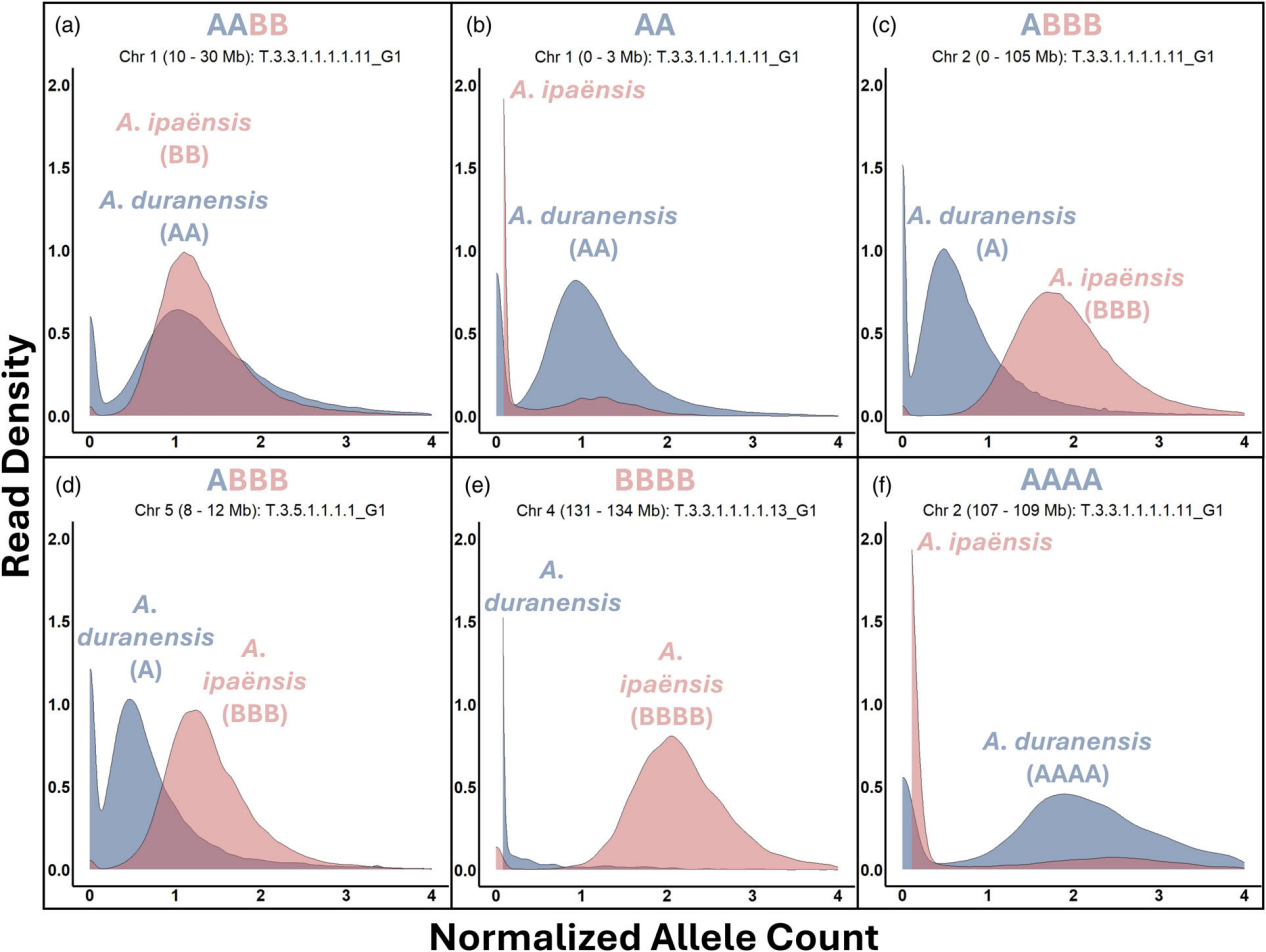
Allele count distributions indicate genome dosage across defined chromosome regions. Each panel shows read density distributions of normalized allele counts within chromosomal windows, based on whole genome sequencing. A subgenome allele counts are shown in blue, and B subgenome counts in red. These profiles illustrate characteristic dosage patterns used to classify genomic composition: (a) Balanced AABB; (b) Deletion at the top of chromosome B01 (AA); (c, d) ABBB; (e) BBBB; (f) AAAA. These distributions support the dosage classifications shown in Figures 2 and 4.

Plants with abnormal genotyping profiles, along with selected progeny, were subjected to whole genome sequencing (WGS). Sequencing of T.3.3.1.1.1.1.11_G1, T.3.3.1.1.1.1.13_G1, and T.3.3.1.1.1.1.14_G1 confirmed that the abnormal genotyping observed in their parent, T.3.3.1.1_G1, was due to a large deletion at the top of chromosome B01. Sequencing of T.3.3.1.1.1.1.11_G1 and T.3.5.1.1.1.1_G2 also confirmed ABBB‐genome compositions on chromosomes A02/B02 and A05/B05, respectively (Figure 4a–c; Figure 5a–f; Additional File 1: Figures S2–S7).

### The deletion on chromosome B01 reduces pod width and seed size

In generation 6, plant T.3.3.1.1.1.1_G1, which carried a deletion at the top of chromosome B01, showed visibly reduced pod width compared to other ‘Tifrunner’ lineages (Additional File 1: Figure S8). To evaluate this effect statistically, 16 progeny of T.3.3.1.1.1.1_G1 were grown in generation 7, alongside 16 progeny of a plant with normal genome composition, T.2.5.1.1.1.1_G1 as a control.

**Figure 6:**
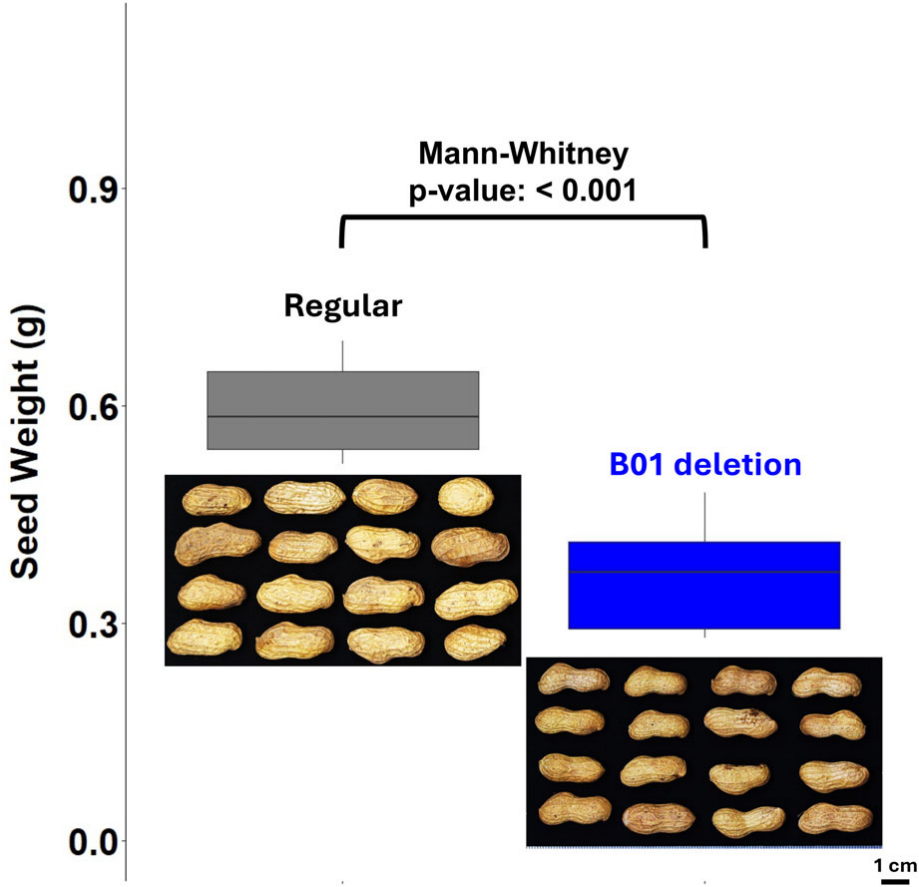
A deletion on chromosome B01 reduces seed weight and pod size. Boxplots compare seed weight between ‘Tifrunner’ plants with a normal genome composition (gray) and those carrying a spontaneous deletion at the top of chromosome B01 (blue). Representative pods are shown below each group. Plants with the B01 deletion produced significantly smaller seeds and narrower pods. Statistical tests: Shapiro–Wilk *P* < 0.001 (non‐normality), Levene *P* = 0.7278 (equal variances), Mann–Whitney *P* < 0.001 (seed weight difference).

Principal component analysis of pod morphology separated the two groups clearly (Figure 3b; Additional File 1: Figure S9), with key differences driven by pod width, minor axis, convexity, minimum radius, and minimum diameter. These traits were all reduced in the deletion‐carrying lineage across both generations (Figure 6; Additional File 1: Figures S10–S12). Seed weight was also significantly lower in generation 7.

These results indicate that the B01 deletion reduces pod size and seed weight, and that its phenotypic effect is stable across generations.

## DISCUSSION

Peanut presents a fascinating paradox. Its recent and singular evolutionary origin less than 10 000 years ago through polyploidization created a genetic bottleneck and isolated peanut from its wild relatives. Despite this, the crop has developed remarkable phenotypic diversity (Krapovickas et al., 2009; Krapovickas & Gregory, 1994; Krapovickas & Gregory, 2007). How was this possible?

Over several years, our curiosity was sparked by infrequent but apparently distinct chromosomal abnormalities appearing across multiple genetic datasets of pure pedigree *A. hypogaea*. Most genotypic analyses filter out unexpected genetic events such as deletions, aneuploidies, and other copy number variants. Even when noticed, these anomalies tend to be dismissed as noise or spurious results, and their sporadic nature makes it inherently difficult to determine their origin or significance. In broad germplasm screens, their origin and timing are unclear, and context is absent. Data from structured populations like recombinant inbred lines (RILs) offer potential but still have very significant uncertainties: heterogeneity in parental lines, seed mixtures, and the charming but genetically indiscriminate pollination by bees all conspire to confound firm conclusions.

Nevertheless, these recurring observations prompted us to make a hypothesis: that spontaneous chromosomal instabilities are surprisingly frequent in *A. hypogaea* of pure pedigree. To test this, we designed a carefully controlled experiment—one that could seem eccentric at first glance—in which ‘Tifrunner’ lineages, all derived from a single highly selfed plant from the ‘genome stock’, were advanced through seven generations of single‐seed descent under pollinator‐free greenhouse conditions. This approach, free from conventional genetic segregation, external gene flow or seed mixture, allowed us to detect instabilities and place them in their proper context with confidence.

The findings supported our hypothesis. Three out of 233 ‘Tifrunner’ plants (1.3%) exhibited novel large‐scale chromosomal instabilities. One of these events had clear, heritable phenotypic consequences: a deletion on chromosome B01 was associated with reduced pod width and seed weight—both key agronomic traits.

**Figure 7:**
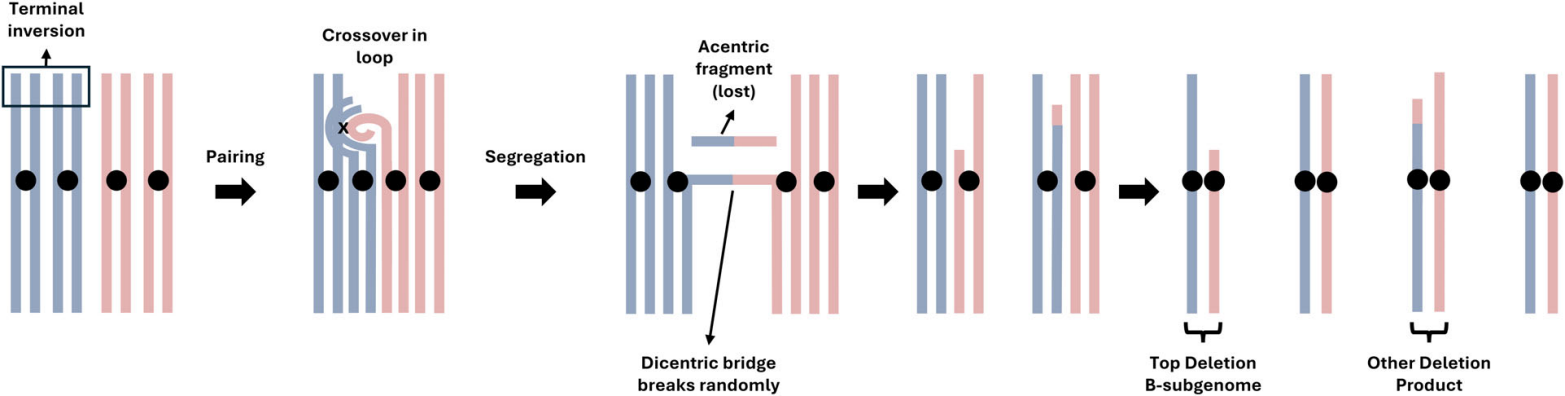
A terminal inversion explains the chromosome B01 deletion via a classic cytogenetic mechanism. The homeologous chromosome A01 (blue) carries a terminal inversion relative to chromosome B01 (red). During homeologous pairing, an inversion loop forms. A crossover within the loop generates a dicentric bridge and an acentric fragment. Upon segregation, the bridge breaks and the acentric fragment is lost, producing a gamete with a terminal deletion on B01. This mechanism, described by Brandham (1977), accounts for the spontaneous deletion observed at the top of chromosome B01.

The observed deletion is best explained by a classical mechanism of meiotic breakage well documented in structural cytogenetics (Figure 7). Pairing between the upper regions of B01 and A01—the latter bearing a large inversion relative to its homeolog (Bertioli et al., 2016)—would form a loop. A crossover within this loop would generate structurally unbalanced chromatids, one of which carries a deletion. This well‐established cytogenetic process (Brandham, 1977) offers a highly plausible explanation for the origin of the B01 deletion.

These findings build on previous documentation of tetrasomic recombination in tetraploid *Arachis* (Bertioli et al., 2016; Bertioli et al., 2019; Chu et al., 2021; Clevenger et al., 2017; Lamon et al., 2025; Leal‐Bertioli et al., 2015; Nguepjop et al., 2016), but show that it is ongoing at a surprising frequency in modern *A. hypogaea* of pure pedigree. The identification of a deletion promoted by a homeologous inversion is also new.

With these carefully controlled ‘Tifrunner’ results in hand, we revisited high‐quality datasets from two other populations and carefully examined them for instabilities that potentially had occurred spontaneously during population development. Although uncertainties remain, what once appeared as isolated anomalies could now be seen with greater confidence as part of a broader, persistent pattern of chromosomal instability in pure pedigree cultivated peanut:

In a recombinant inbred line (RIL) population comprising 166 individuals (Zhou et al., 2014), we identified nine plants exhibiting chromosomal abnormalities. These were detected using normalized RAD‐seq mapping densities, which allowed inference of dosage variation across the genome. These included two individuals with supernumerary chromosome configurations: one with an AABBB composition for chromosome set 01, and another with an AAABB composition for chromosome set 05. To our knowledge, these represent the best‐defined cases of supernumerary chromosomes in pure pedigree *A. hypogaea*, and they mirror similar events observed in the neotetraploid IpaDur1 (Lamon et al., 2025). Although some cytogenetic studies have reported extra chromosomes (e.g., Husted, 1936), such observations are much less precise because of the ambiguous nature of the extra chromosome and the limitations inherent to chromosome squashes. One RIL carried a terminal deletion on the lower portion of chromosome A07, and two others carried large deletions at the start of chromosome A08 (Additional File 3: Tables S7 and S8). Notably, A07 and A08 are derived from reciprocal translocations and inversions involving B07 and B08 (Bertioli et al., 2016) suggesting that these deletions may be further examples of homeologous inversion‐mediated breakage. Taken together, these nine individuals with clear chromosomal abnormalities indicate a frequency of instability of 5.4% in this RIL population.

In a second RIL population derived from a cross between ‘Tifrunner’ and a germplasm line NC 3033 (Chavarro et al., 2020; Holbrook et al., 2013), we identified four distinct chromosomal instabilities among 202 individuals. These were inferred from allele dosage genotyping calls using the ‘fitPoly’ package (Voorrips, 2025; Voorrips et al., 2011), which allowed detection of dosage variation across the genome. One—a small possible deletion in chromosome set 01—was shared by 35 individuals and seems likely to reflect heterogeneity in the parents used to create the population, so we excluded it from consideration. Another, affecting chromosome set 03, was observed in six individuals in both AAAB and AAAA forms, which seems consistent with instability during line development. The remaining two were unique to individual lines (Additional File 4: Tables S9 and S10). Depending on how the chromosome 03 event is interpreted—excluded entirely, counted as a single instability, or considered six independent events—the estimated incidence of spontaneous instability ranges from 1.0% to 4.0%.

While this level of instability in *A. hypogaea* is surprising, it is markedly lower than that observed in the neoallotetraploid derived from *A. ipaënsis* and *A. duranensis*, IpaDur1. In the early‐generation polyploids, large‐scale genomic instabilities are frequent, even though their component subgenomes are strikingly similar to those of *A. hypogaea*. This suggests that the relative genetic stability arises not from differences in structure and inherent chromosome affinities, but from differences in regulation. This interpretation is consistent with foundational work in other polyploid crops where genetic suppression of homeologous pairing has long been recognized (Martín et al., 2017; Riley & Chapman, 1958; Szadkowski et al., 2010) and is supported by work in peanut from Leal‐Bertioli et al. (2015), who used a population combining cultivated and wild genetics to map QTLs associated with genome instability. The relatively stable disomic inheritance of *A. hypogaea*, in contrast to the unstable, highly segmental behavior of neoallotetraploids, likely reflects the accumulation and fixation of alleles that suppress homeologous pairing. In this context, residual instability in the modern crop may reflect incomplete or leaky suppression of those interactions and may vary substantially with genetic background.

Despite the leaky suppression of homeologous recombination in *A. hypogaea*, the fixation of large new BBBB and AAAA regions or deletions is likely deleterious for two reasons: they reduce peanut's fixed hybrid vigor and compromise genome stability. Large tetrasomies erode the fixed AABB hybrid balance and increase homeologous affinity, promoting mispairing. Deletions likewise disrupt the hybrid state and may also unmask harmful alleles. These patterns align with the ‘polyploid ratchet’ model, in which structural changes accumulate and gradually diminish the genome's capacity to return to stability (Gaeta & Pires, 2010). Selection, therefore, likely limits the persistence of most changes that undermine the fixed hybrid state.

Because these large‐scale genome changes are usually purged by selection, they might be dismissed as transient and unimportant. However, like mutations, a small proportion presumably confer advantages and are selected. Also, we suggest that ABBB‐ or AAAB‐genome compositions may drive more subtle but lasting consequences. In diploid organisms, homologous chromosomes guide accurate DNA repair. Compositions such as ABBB and AAAB leave chromosomal regions orphaned, lacking a true homolog. In such cases, the use of the homeologous chromosome for repair would promote gene conversion and other forms of homeologous flux. Given the observed frequency of these configurations in pure pedigree cultivated peanut, this mechanism may act often enough to shape long‐term genome evolution by promoting finer‐scale ongoing exchanges between the A‐ and B‐subgenomes.

This paper concludes our trilogy elucidating the origin and evolution of peanut. Our first paper reaffirmed the narrow origin of *A. hypogaea* from a single hybridization. The second showed how even a single polyploidization event can give rise to a boom of genome instability and phenotypic diversity, and showed an improved response to artificial selection—a polyploid advantage for peanut domestication. The third demonstrated that chromosomal instability persists to a surprising degree even in homozygous lineages of pure pedigree *A. hypogaea*, such that it likely continues to shape the evolution of this remarkable crop.

## MATERIALS & METHODS

### Plant material

Plants to be used in this study were: the accessions of the peanut progenitors *A. duranensis* V 14167 (PI 692197) and *A. ipaënsis* K 30076 (PI 468322), and the highly selfed ‘genome stock’ of the modern cultivar *A. hypogaea* subsp. *hypogaea* ‘Tifrunner’ (registration number CV‐93, PI 644011). A single seed of ‘Tifrunner’ was planted in a pollinator‐free greenhouse in 2019. In generation 1 (2020), six seeds from this ‘Tifrunner’ plant (‘T’) were cultivated in two separate greenhouses: three in greenhouse 1 (‘G1’) and three in greenhouse 2 (‘G2’). These ‘Tifrunner’ plants were advanced through six additional generations via selfing, by single‐seed descent.

Two selected plants from generation 6, namely T.2.5.1.1.1.1_G1 and T.3.3.1.1.1.1_G1, were selected to be expanded to 16 plants each, rather than continuing with single‐seed descent. T.3.3.1.1.1.1_G1 was selected because it exhibited a noticeable reduction in pod width, prompting an investigation into the heritability of this trait. T.2.5.1.1.1.1_G1, which exhibited regular seed and pod dimensions, was arbitrarily selected as a control. This change in advancement strategy was implemented to collect the necessary phenotypic traits and obtain sufficient measurements for statistical analysis, allowing for a determination of whether the differences between T.2.5.1.1.1.1_G1 and T.3.3.1.1.1.1_G1 were statistically significant.

The remaining plants continued under single‐seed descent until generation 7, yielding a total of 62 surviving lineages. In total, 233 plants were derived from the original T plant across seven generations (Figure 1).

### Genotyping and sequencing

DNA was extracted from young leaves of all ‘Tifrunner’ plants using the DNeasy Plant Mini Kit (Qiagen, Hilden, Germany). Control samples included three samples of *A. duranensis* V 14167 and four of *A. ipaënsis* K 30076, mixtures each at ratios of 1:1, 3:1, and 1:3 of *A. duranensis* to *A. ipaënsis*, representing AABB‐, AAAB‐, and ABBB‐genome compositions, respectively. Generation 1 plants (T.1_G1, T.2_G1, T.3_G1, T.1_G2, T.2_G2, and T.3_G2) were extracted twice for use as controls. Genotyping was performed using the Axiom *Arachis* 48 K SNP array v2 (Affymetrix, Santa Clara, CA, USA) (Clevenger et al., 2017; Korani et al., 2019; Pandey et al., 2017). Additionally, T.3.3.1.1.1.1.11_G1, T.3.3.1.1.1.1.13_G1, T.3.3.1.1.1.1.14_G1, T.3.5.1.1.1.1_G2, and T.3.5.1.1.1.1.1_G2 were sequenced at 30x coverage using Illumina WGS NovaSeq (PE150) technology (Novogene Co, Sacramento, CA, USA) (Figure 1).

### Genotypic data analysis

Genotypic data was analyzed in RStudio (version R‐4.2.2) (R Core Team, 2023) and followed a procedure similar to the one outlined in Lamon et al. (2025). SNP markers were scored on a tetraploid scale (0 to 4), representing the potential allele dosages found in segmental allotetraploid peanuts. This scoring system was applied to all samples, including the diploid progenitors *A. duranensis* and *A. ipaënsis*. The scoring utilized the ‘fitPoly’ package (Additional file 2: Table S2).

The dataset underwent multiple filtering steps to enhance its quality. Initially, markers with complete scoring across all lineages were retained. Markers were further selected based on their consistency with expected scoring patterns in different genome compositions. Specifically, markers were retained if control groups (*A. duranensis*, *A. ipaënsis*, 1:1 DNA mixtures [AABB], 3:1 DNA mixtures [AAAB], and 1:3 DNA mixtures [ABBB]) demonstrated uniform values. Markers were also kept if *A. duranensis* scores differed from *A. ipaënsis* scores by at least two decimal places (e.g., *A. duranensis* = 3, *A. ipaënsis* = 1), 3:1 mixtures (AAAB) differed from 1:3 mixtures (ABBB), and 1:1 mixtures (AABB) had a modal score of 2, indicating subgenome homozygosity for opposing alleles.

Further refinement involved filtering markers to ensure redundant samples from generation 1 plants had matching scores to exclude low‐quality markers. Markers were retained if the modal score for ‘Tifrunner’ plants was 2 or if two or more consecutive markers showed a modal score deviating from 2, leaning towards *A. duranensis* (scores 3 or 4) or *A. ipaënsis* (scores 0 or 1). This step helped eliminate mis‐scored markers while preserving markers indicating unbalanced genome compositions across several ‘Tifrunner’ lineages. The filtration process prioritized markers aligned with the physical positions of the *A. ipaënsis* gnm1 genome, reducing the dataset to 1384 markers.

Scores were standardized by converting markers so that *A. duranensis* alleles were represented by higher modal values, effectively counting A alleles (i.e., BBBB = 0, ABBB = 1, AABB = 2, AAAB = 3, AAAA = 4). This standardization was confirmed as 3:1 DNA mixtures (AAAB) consistently displayed higher modal scores than 1:3 DNA mixtures (ABBB). Two ‘Tifrunner’ plants were excluded due to failing Affymetrix quality filters. Four more were removed for having many non‐modal scores, likely from low‐quality DNA. The final dataset includes 227 ‘Tifrunner’ plants (Additional file 2: Table S3).

A color‐coded matrix was generated using ‘ggplot2’ (Wickham, 2025) based on the physical positions of *A. ipaënsis* gnm1 chromosomes to visualize genome compositions among ‘Tifrunner’ plants, highlighting BBBB, ABBB, AAAB, and AAAA configurations within chromosome sets. To further investigate genetic relationships among ‘Tifrunner’ lineages, PCA was conducted using ‘adegenet’ (Jombart & Ahmed, 2011), ‘ade4’ (Dray et al., 2007), ‘FactoMineR’ (Lê et al., 2008) and ‘factoextra’ (Kassambara & Mundt, 2020).

Novel large‐scale genetic instability was evaluated across generations by identifying the spontaneous appearance of abnormal genetic states. Rates were calculated based on the total number of plants analyzed, and trends were summarized to assess changes over time.

### Sequencing data analysis

Sequencing data analysis as well followed the procedure described by Lamon et al. (2025). Briefly, DNA from both *A. duranensis* (AA‐genome) and *A. ipaënsis* (BB‐genome) was fragmented into 10 kb genome fragments. Additionally, Illumina sequencing data were obtained from two independent sources: Beijing Genomics Institute and HudsonAlpha. Quality filtering was performed using BBMap (version 39.01) (Bushnell, 2014).

Filtered reads were aligned to the *A. ipaënsis* gnm1 reference genome using BWA (version 0.7.17‐r1188) (Li & Durbin, 2009), followed by variant calling with BCFtools (version 1.15.1) (Danecek et al., 2021). Target AB variant positions were identified based on specific filtering criteria, including agreement between genome fragments and Illumina reads, 95% consensus of mapped reads, a minimum depth of 1 for genome fragments, and differing alleles between A‐ and B‐subgenomes.

T.3.3.1.1.1.1.11_G1, T.3.3.1.1.1.1.13_G1, T.3.3.1.1.1.1.14_G1, T.3.5.1.1.1.1_G2, and T.3.5.1.1.1.1.1_G2, as well as the ‘Tifrunner’ reference genome (registration number CV‐93, PI 644011) (Bertioli et al., 2019), were aligned to the *A. ipaënsis* reference genome for genetic variants analysis. To normalize allele counts, synthetic tetraploid peanut reads were generated by combining WGS reads from HudsonAlpha in a 1:1 ratio of A‐ and B‐subgenomes. These synthetic reads were mapped to the reference genome, and genetic variants at target positions were identified. Then, normalization of raw allele counts was performed for A‐ and B‐subgenomes to ensure accurate comparisons across samples. Normalized allele counts for the ‘Tifrunner’ plants were visualized in density plots created using the ‘ggplot2’, and comparisons with genotypic data were summarized using ‘chromoMap’ (Anand & Rodriguez Lopez, 2022).

### Phenotypic data collection and analysis

Phenotypic data collection and analysis followed the procedure outlined by Lamon et al. (2025). Pod traits including pod area, pod area convex hull, pod perimeter, pod mean radius, pod minimum radius, pod maximum radius, pod radius standard deviation, pod mean diameter, pod minimum diameter, pod maximum diameter, pod major and minor axes, pod caliper, pod length, pod width, pod radius ratio, pod eccentricity, pod form factor, pod narrow factor, pod aspect ratio, pod rectangularity, pod perimeter–caliper ratio, pod perimeter–length–width ratio, pod solidity, pod convexity, pod elongation, pod circularity, pod Haralick circularity, pod normalized circularity, and number of pods (Additional file 2: Table S4) were measured for all ‘Tifrunner’ plants in generation 6 and selected plants in generation 7 (Olivoto, 2022) (Additional file 2: Table S5). The analyzed generation 7 plants, derived from T.2.5.1.1.1.1_G1 and T.3.3.1.1.1.1_G1, included plants T.2.5.1.1.1.1.12_G1 to T.2.5.1.1.1.1.16_G1 and T.3.3.1.1.1.1.12_G1 to T.3.3.1.1.1.1.16_G1. Additionally, seed weights were recorded for T.2.5.1.1.1.1.2_G1 to T.2.5.1.1.1.1.11_G1 and T.3.3.1.1.1.1.2_G1 to T.3.3.1.1.1.1.11_G1 plants (Additional file 2: Table S6).

Pod characteristics were recorded by photographing pods against a dark background using a Canon EOS Rebel T3 camera with a Canon Macro Lens EF‐S 60 mm (Canon Inc., Tokyo, Japan). Images were taken from a lateral view to capture the pod profile, typically placing the residual peg in one of the four image corners. Image processing was performed with ‘pliman’ (Olivoto, 2022). Before analysis, images were edited in Paint 3D (Microsoft, Redmond, WA, USA) to remove unwanted elements and then imported into RStudio. Pod characteristics were extracted using the analyze_objects() function. Measurements were calibrated to the image's known dpi, which was calculated using the dpi() function on a ruler in the image via the get_measures() function.

A PCA was performed on pod traits using the ‘adegenet’, ‘ade4’, ‘FactoMineR’, and ‘factoextra’ packages. The contributions of variables to the first five principal components were visualized with ‘corrplot’ (Wei & Simko, 2017). Pod width, pod minor axis, pod minimum radius, and pod minimum diameter were tested for normality using the Shapiro–Wilk test and for homoscedasticity with Levene's test, employing functions from the ‘stats’ and ‘car’ packages. Kruskal–Wallis tests were performed using the ‘stats’ package, followed by Dunn's multiple comparisons from the ‘FSA’ package (Ogle et al., 2025). Results were presented with a compact letter display using the ‘rcompanion’ package (Mangiafico, 2025). Similarly, seed weight analysis followed the same initial testing for normality and homoscedasticity as the pod traits. However, comparisons were made using a Mann–Whitney U test, with *P*‐values reported directly. Data visualization for both pod traits and seed weight was done using ‘ggplot2’.

## AUTHOR CONTRIBUTIONS

DJB and SCML‐B conceptualized the project. SL collected phenotypic data, analyzed both phenotypic and genotypic data, and visualized the sequencing data. BLA and DJB conducted the analysis of sequencing data and contributed to its visualization. The manuscript was written by SL and DJB. The manuscript was approved by all authors.

## CONFLICT OF INTEREST

The authors declare no conflict of interest.

## Supporting information


**Figure S1.** Scree plot of genotypic principal component analysis of ‘Tifrunner’ plants.
**Figure S2.** Density plots of allele counts across chromosome sets in ‘Tifrunner’ peanut genome (CV‐93, PI 644011).
**Figure S3.** Density plots of allele counts across chromosome sets in T.3.3.1.1.1.1.11_G1.
**Figure S4.** Density plots of allele counts across chromosome sets in T.3.3.1.1.1.1.13_G1.
**Figure S5.** Density plots of allele counts across chromosome sets in T.3.3.1.1.1.1.14_G1.
**Figure S6.** Density plots of allele counts across chromosome sets in T.3.5.1.1.1.1_G2.
**Figure S7.** Density plots of allele counts across chromosome sets in T.3.5.1.1.1.1.1_G2.
**Figure S8.** Photographic images of generation 6 ‘Tifrunner’ plants cultivated in greenhouse 1 and 2.
**Figure S9.** Scree plot of principal component analysis for pod traits in generation 6 and 7 ‘Tifrunner’.
**Figure S10.** Contribution of pod phenotypic variables to the first five dimensions of principal component analysis in ‘Tifrunner’ lineages.
**Figure S11.** Contribution and correlation of phenotypic traits in principal component analysis of ‘Tifrunner’ pod data.
**Figure S12.** ‘Tifrunner’ lineage with a deletion at the top of chromosome B01 shows significantly lower pod trait values.

**Table S1.** Genetic instability rate.
**Table S2.** Genotyping calls.
**Table S3.** Final matrix.
**Table S4.** Overview of the collected pods phenotypic data of ‘Tifrunner’ in generation 6 and 7.
**Table S5.** Collected pods traits.
**Table S6.** Collected seed weight trait.

**Table S7.** Zhou et al RIL RAD‐seq mapping densities versus P2.
**Table S8.** Zhou et al RIL RAD‐seq mapping densities versus P1.

**Table S9.** ‘Tifrunner’ x NC3033 RIL genotyping.
**Table S10.** ‘Tifrunner’ x NC3033 RIL WGS mapping densities.

## Data Availability

WGS data can be accessed in NCBI under BioProject accession PRJNA1238928 and PRJNA1274041. All other data generated or analyzed during this study are included in the additional files. Sequencing analysis scripts are available at https://github.com/brianabernathy/ABGD.
