# Supplementary material for: Surprisingly frequent chromosomal instability in cultivated peanut
Source: Plant J. 2025 Dec 24;124(6):e70617. doi: 10.1111/tpj.70617 (PMC12737953; doi:10.1111/tpj.70617)
Supplement: Supplementary file 1 — Figure S1. Scree plot of genotypic principal component analysis of ‘Tifrunner’ plants. Figure S2. Density plots of allele counts across chromosome sets in ‘Tifrunner’ peanut genome (CV‐93, PI 644011). Figure S3. Density plots of allele counts across chromosome sets in T.3.3.1.1.1.1.11_G1. Figure S4. Density plots of allele counts across chromosome sets in T.3.3.1.1.1.1.13_G1. Figure S5. Density plots of allele counts across chromosome sets in T.3.3.1.1.1.1.14_G1. Figure S6. Density plots of allele counts across chromosome sets in T.3.5.1.1.1.1_G2. Figure S7. Density plots of allele counts across chromosome sets in T.3.5.1.1.1.1.1_G2. Figure S8. Photographic images of generation 6 ‘Tifrunner’ plants cultivated in greenhouse 1 and 2. Figure S9. Scree plot of principal component analysis for pod traits in generation 6 and 7 ‘Tifrunner’. Figure S10. Contribution of pod phenotypic variables to the first five dimensions of principal component analysis in ‘Tifrunner’ lineages. Figure S11. Contribution and correlation of phenotypic traits in principal component analysis of ‘Tifrunner’ pod data. Figure S12. ‘Tifrunner’ lineage with a deletion at the top of chromosome B01 shows significantly lower pod trait values. [file TPJ-124-0-s001.docx]

**Supplementary Figures for**

**Surprisingly Frequent Chromosomal Instability**

**in Cultivated Peanut**

Samuele Lamon^1^ ([Samuele.Lamon@uga.edu](mailto:Samuele.Lamon@uga.edu); ORCID: [0000-0002-8475-7785](https://orcid.org/0000-0002-8475-7785)), Brian Abernathy^2^ ([bla@uga.edu](mailto:bla@uga.edu)), Soraya C. M. Leal-Bertioli^1,2,3^ ([sbertioli@uga.edu](mailto:sbertioli@uga.edu); ORCID: [0000-0002-9683-5357](https://orcid.org/0000-0002-9683-5357)) and David J. Bertioli^1,2,4^ ([bertioli@uga.edu](mailto:bertioli@uga.edu); ORCID: [0000-0003-0294-7284](https://orcid.org/0000-0003-0294-7284))

^1^Institute of Plant Breeding, Genetics and Genomics, University of Georgia, Athens, GA 30602, USA;

^2^Center for Applied Genetic Technologies, University of Georgia, Athens, GA 30602, USA;

^3^Department of Plant Pathology, University of Georgia, Athens, GA 30602, USA;

^4^Department of Crop & Soil Sciences, University of Georgia, Athens, GA 30602, USA;

Corresponding author: David J. Bertioli.

**
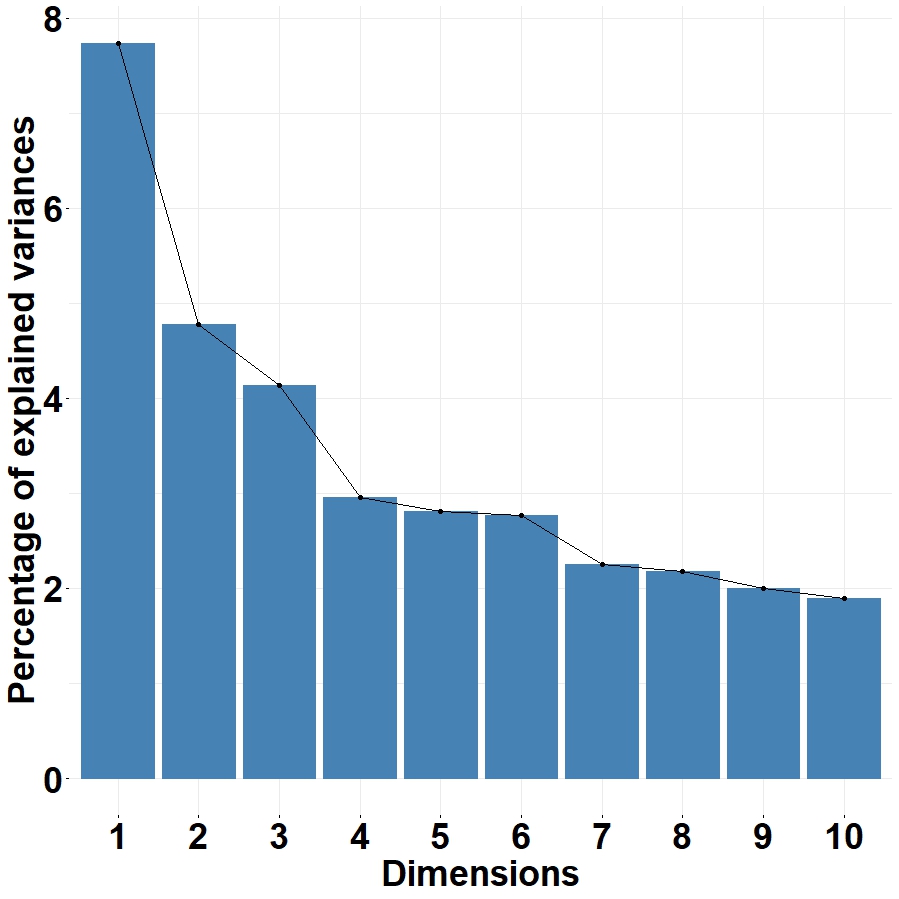
**

**Figure S1: Scree plot of genotypic principal component analysis of ‘Tifrunner’ plants.** The first two principal components account for the majority of the variability, explaining 8% and 5%, respectively.

**
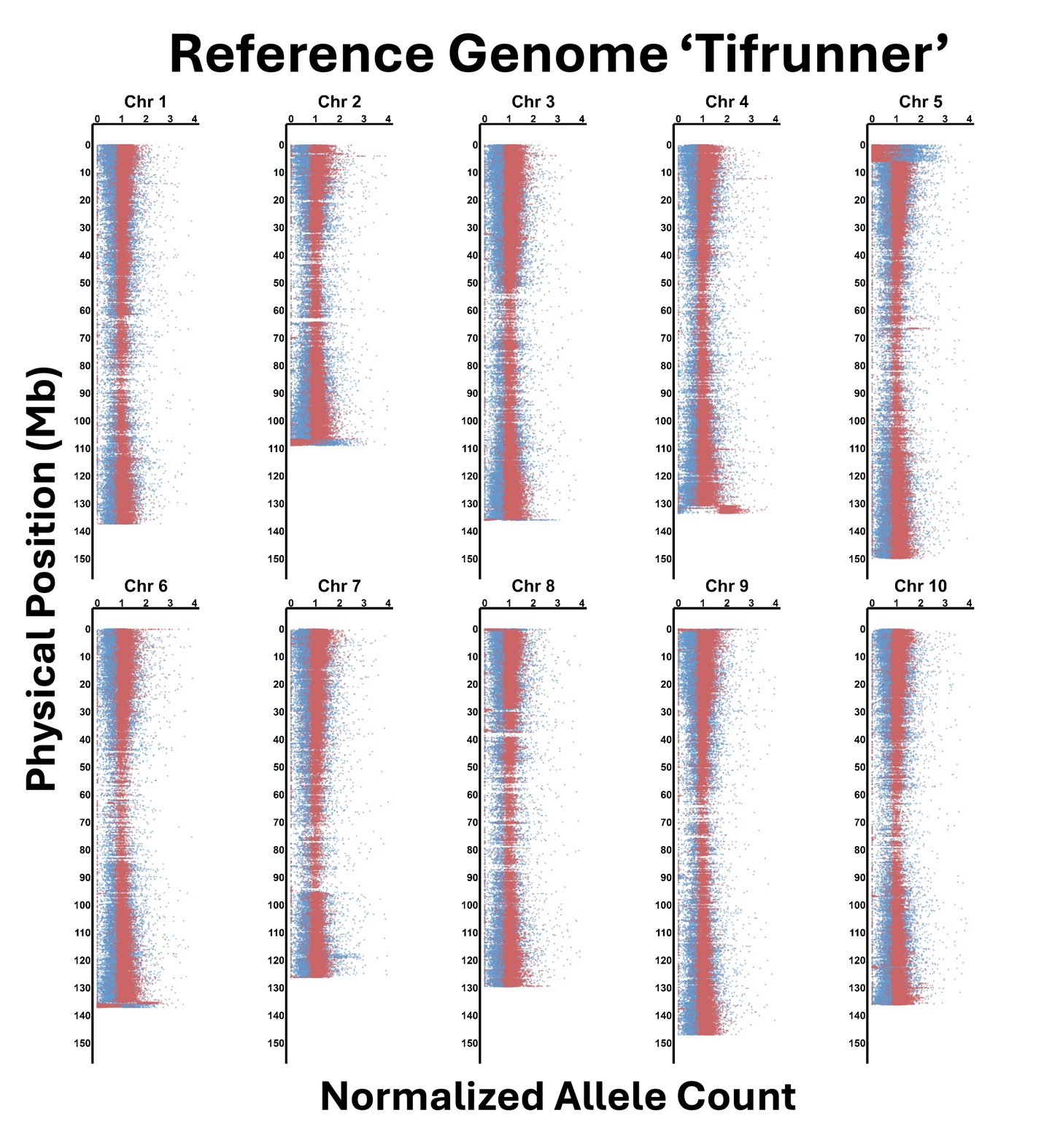
**

**Figure S2: Density plots of allele counts across chromosome sets in ‘Tifrunner’ peanut genome (CV-93, PI 644011).** *Arachis duranensis* alleles are in blue, and *A. ipaënsis* allele counts are in red. Balanced genomic compositions (AABB) are identified by equal counts, while deviations indicate unbalanced genome compositions: BBBB (A = 0, B = 2), ABBB (A = 0.5, B = 1.5), AAAB (A = 1.5, B = 0.5), and AAAA (A = 2, B = 0).

**
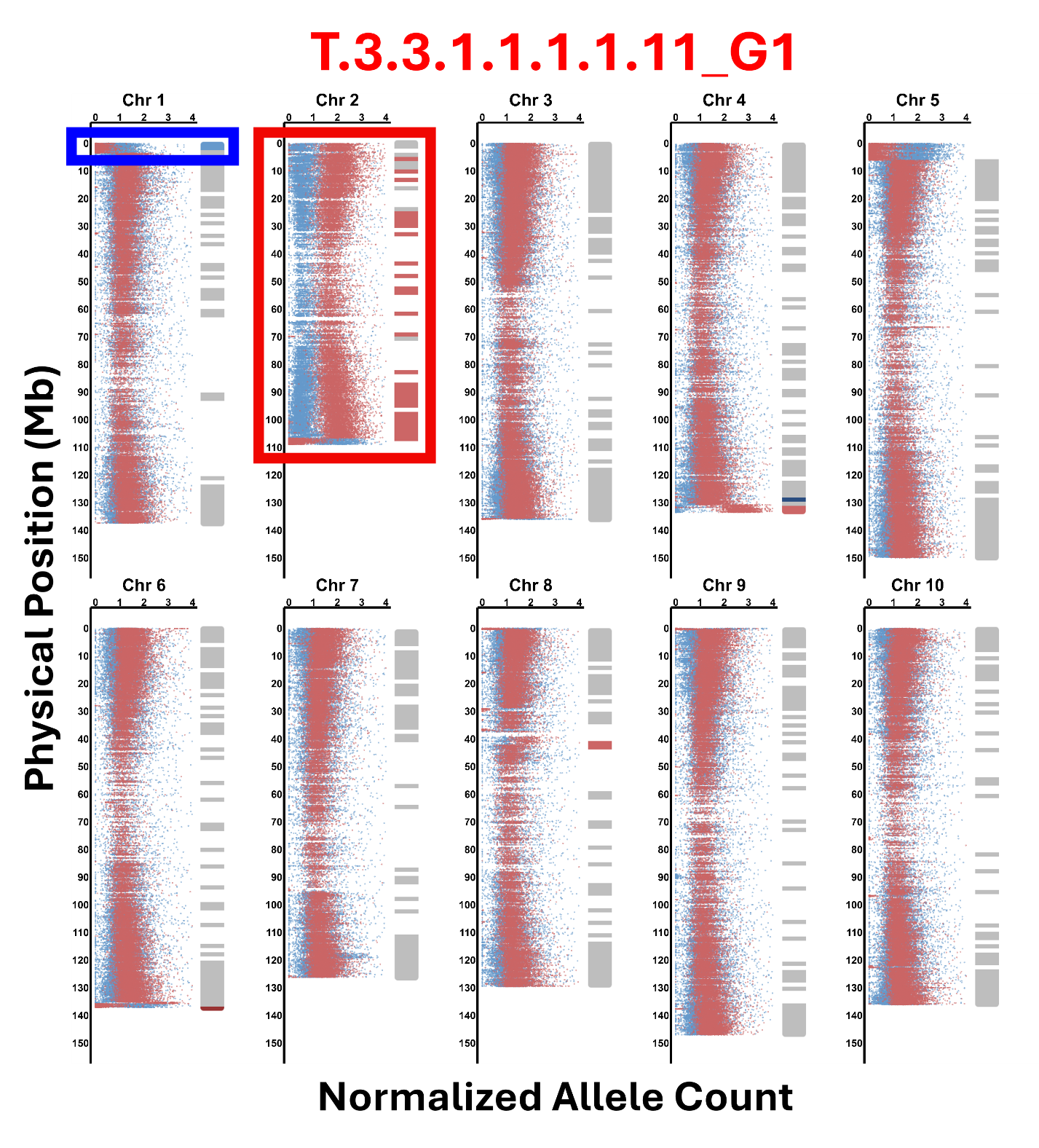
**

**Figure S3: Density plots of allele counts across chromosome sets in T.3.3.1.1.1.1.11_G1.** T.3.3.1.1.1.1.11_G1 is characterized by a B-subgenome deletion on chromosome set 01 and a ABBB genome composition across chromosome set 02. *Arachis duranensis* alleles are in blue, and *A. ipaënsis* allele counts are in red. Balanced genomic compositions (AABB) are identified by equal counts, while deviations indicate unbalanced genome compositions: BBBB (A = 0, B = 2), ABBB (A = 0.5, B = 1.5), AAAB (A = 1.5, B = 0.5), and AAAA (A = 2, B = 0). The red box highlights the ABBB composition across chromosome set 02. The blue box indicates the B-subgenome deletion at the top of chromosome set 01.

**
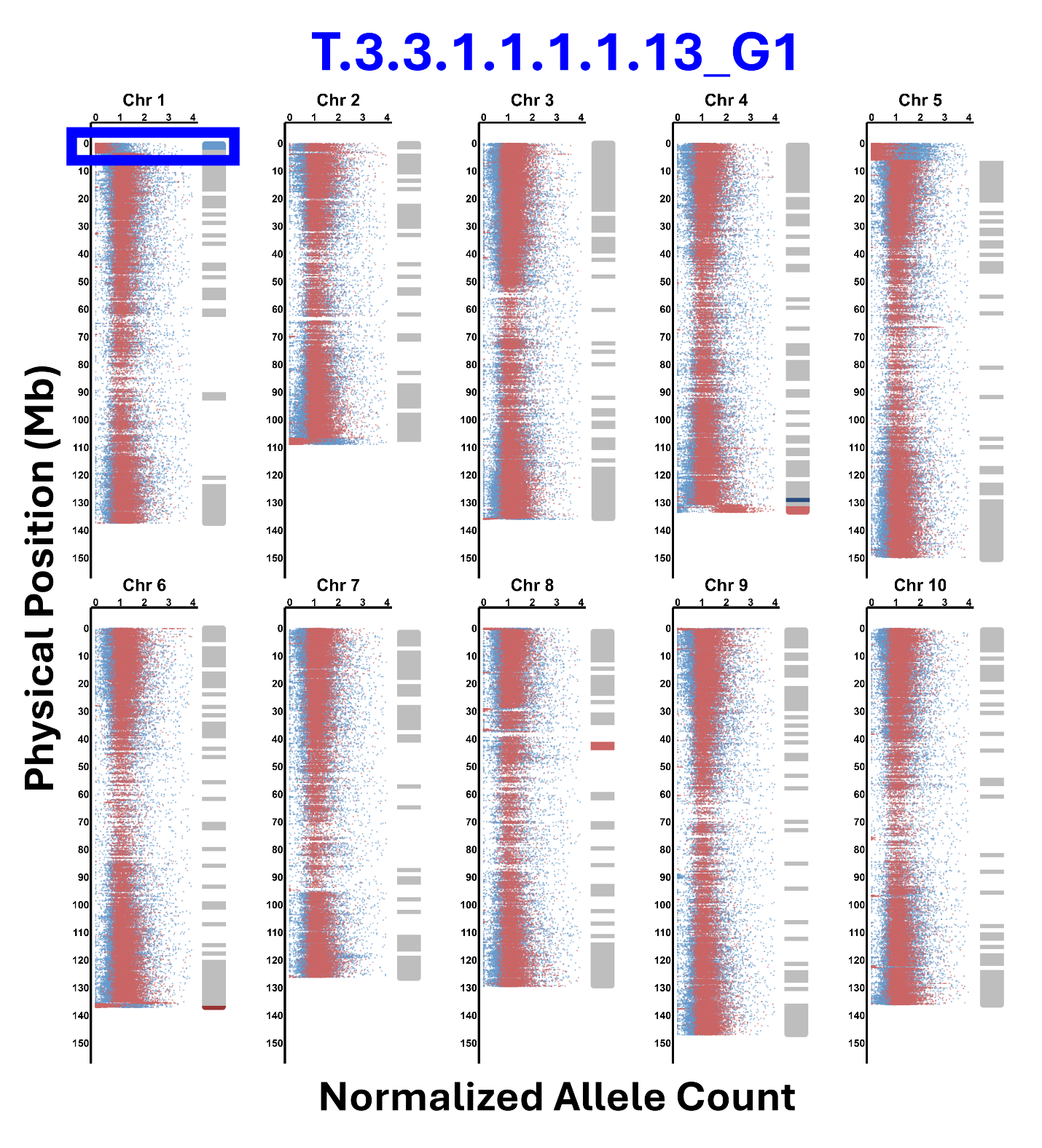
**

**Figure S4: Density plots of allele counts across chromosome sets in T.3.3.1.1.1.1.13_G1.** T.3.3.1.1.1.1.13_G1 is characterized by a B-subgenome deletion on top of chromosome set 01. *Arachis duranensis* alleles are in blue, and *A. ipaënsis* allele counts are in red. Balanced genomic compositions (AABB) are identified by equal counts, while deviations indicate unbalanced genome compositions: BBBB (A = 0, B = 2), ABBB (A = 0.5, B = 1.5), AAAB (A = 1.5, B = 0.5), and AAAA (A = 2, B = 0). The blue box indicates the B-subgenome deletion at the top of chromosome set 01.

**
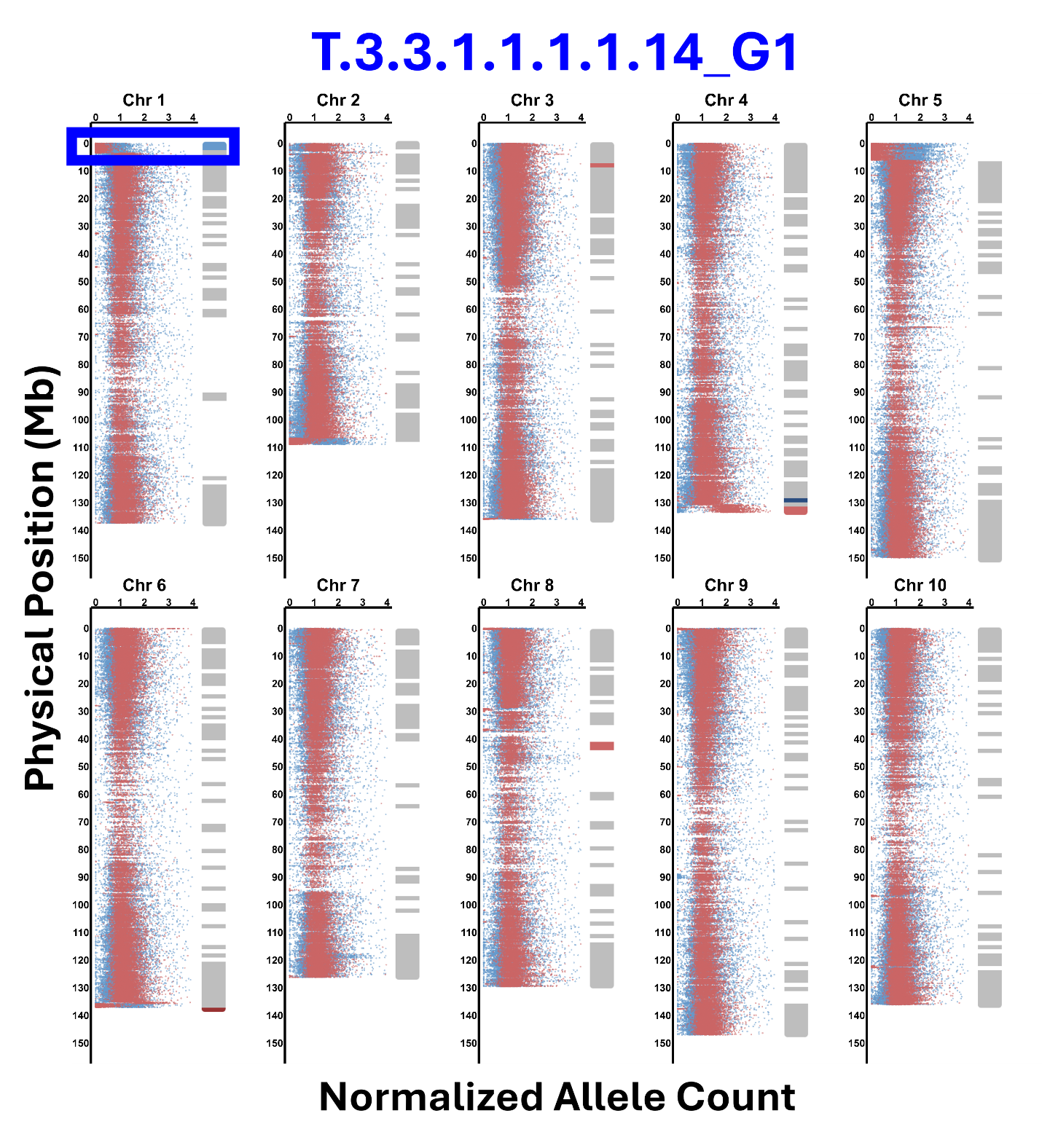
**

**Figure S5: Density plots of allele counts across chromosome sets in T.3.3.1.1.1.1.14_G1.** T.3.3.1.1.1.1.14_G1 is characterized by a B-subgenome deletion on top of chromosome set 01. *Arachis duranensis* alleles are in blue, and *A. ipaënsis* allele counts are in red. Balanced genomic compositions (AABB) are identified by equal counts, while deviations indicate unbalanced genome compositions: BBBB (A = 0, B = 2), ABBB (A = 0.5, B = 1.5), AAAB (A = 1.5, B = 0.5), and AAAA (A = 2, B = 0). The blue box indicates the B-subgenome deletion at the top of chromosome set 01.

**
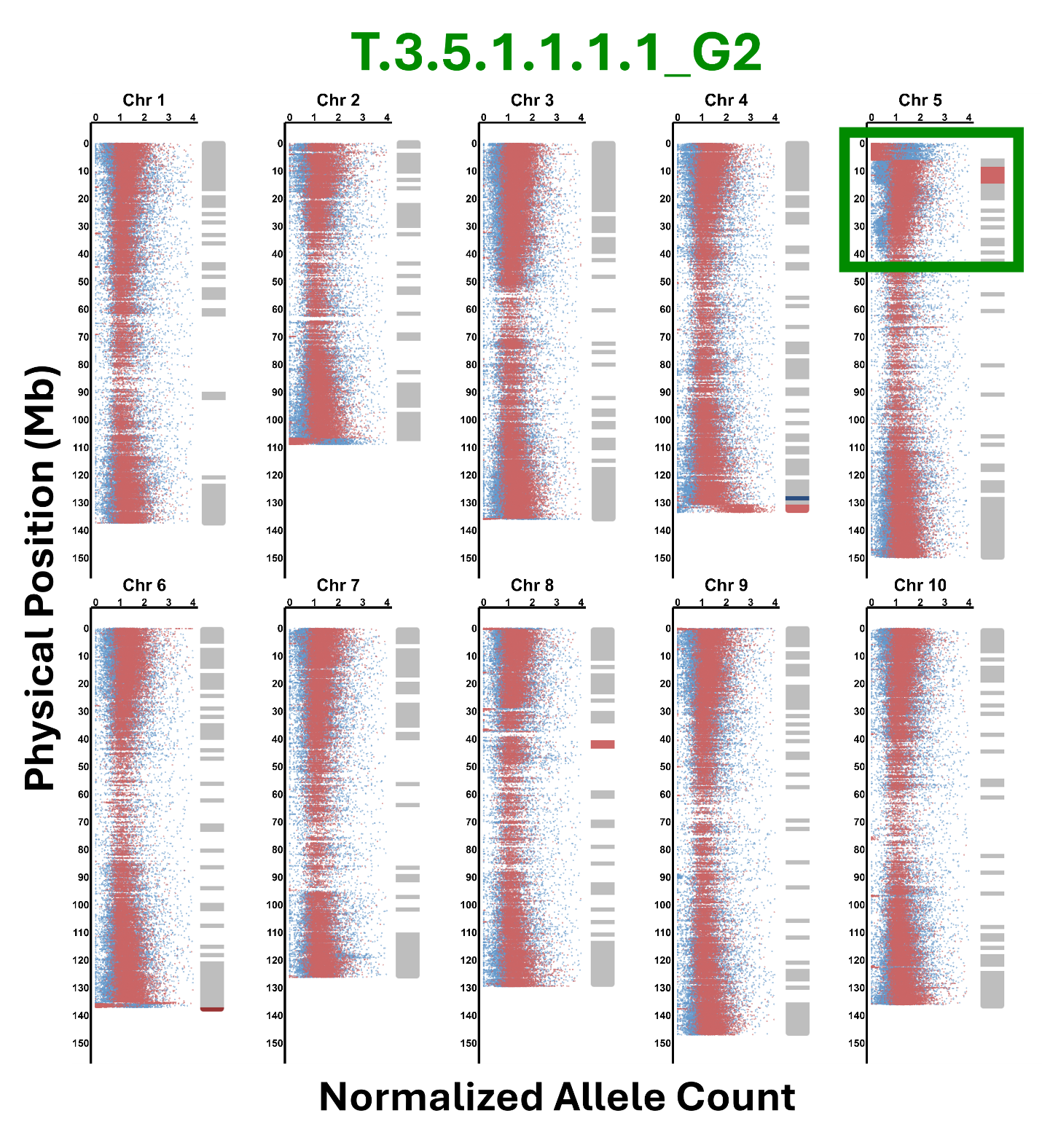
**

**Figure S6: Density plots of allele counts across chromosome sets in T.3.5.1.1.1.1_G2.** T.3.5.1.1.1.1_G2 is characterized by a ABBB genome composition on chromosome set 05. *Arachis duranensis* alleles are in blue, and *A. ipaënsis* allele counts are in red. Balanced genomic compositions (AABB) are identified by equal counts, while deviations indicate unbalanced genome compositions: BBBB (A = 0, B = 2), ABBB (A = 0.5, B = 1.5), AAAB (A = 1.5, B = 0.5), and AAAA (A = 2, B = 0). The green box marks the ABBB composition at the top of chromosome set 05.

**
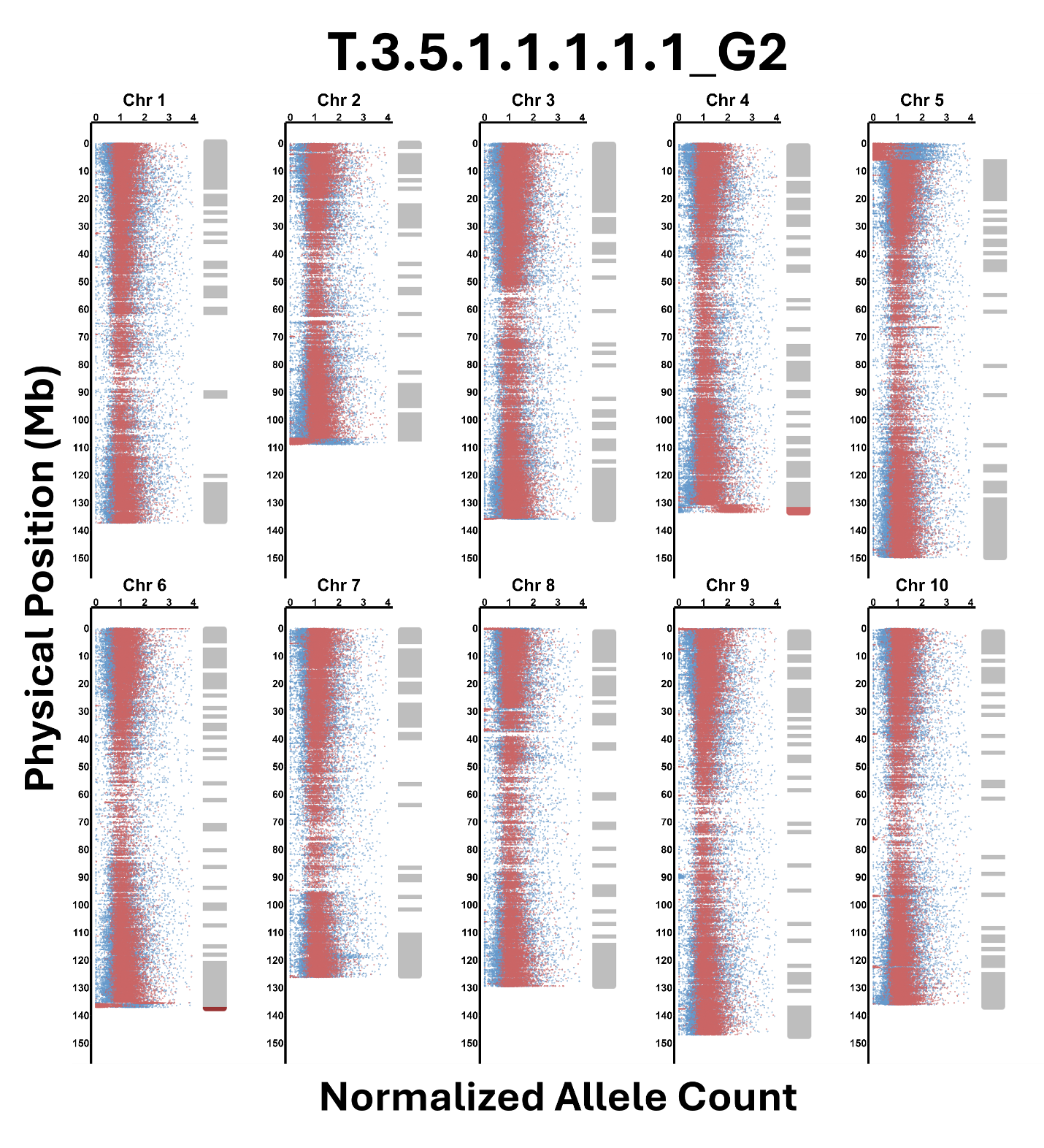
**

**Figure S7: Density plots of allele counts across chromosome sets in T.3.5.1.1.1.1.1_G2.** *Arachis duranensis* alleles are in blue, and *A. ipaënsis* allele counts are in red. Balanced genomic compositions (AABB) are identified by equal counts, while deviations indicate unbalanced genome compositions: BBBB (A = 0, B = 2), ABBB (A = 0.5, B = 1.5), AAAB (A = 1.5, B = 0.5), and AAAA (A = 2, B = 0).

**
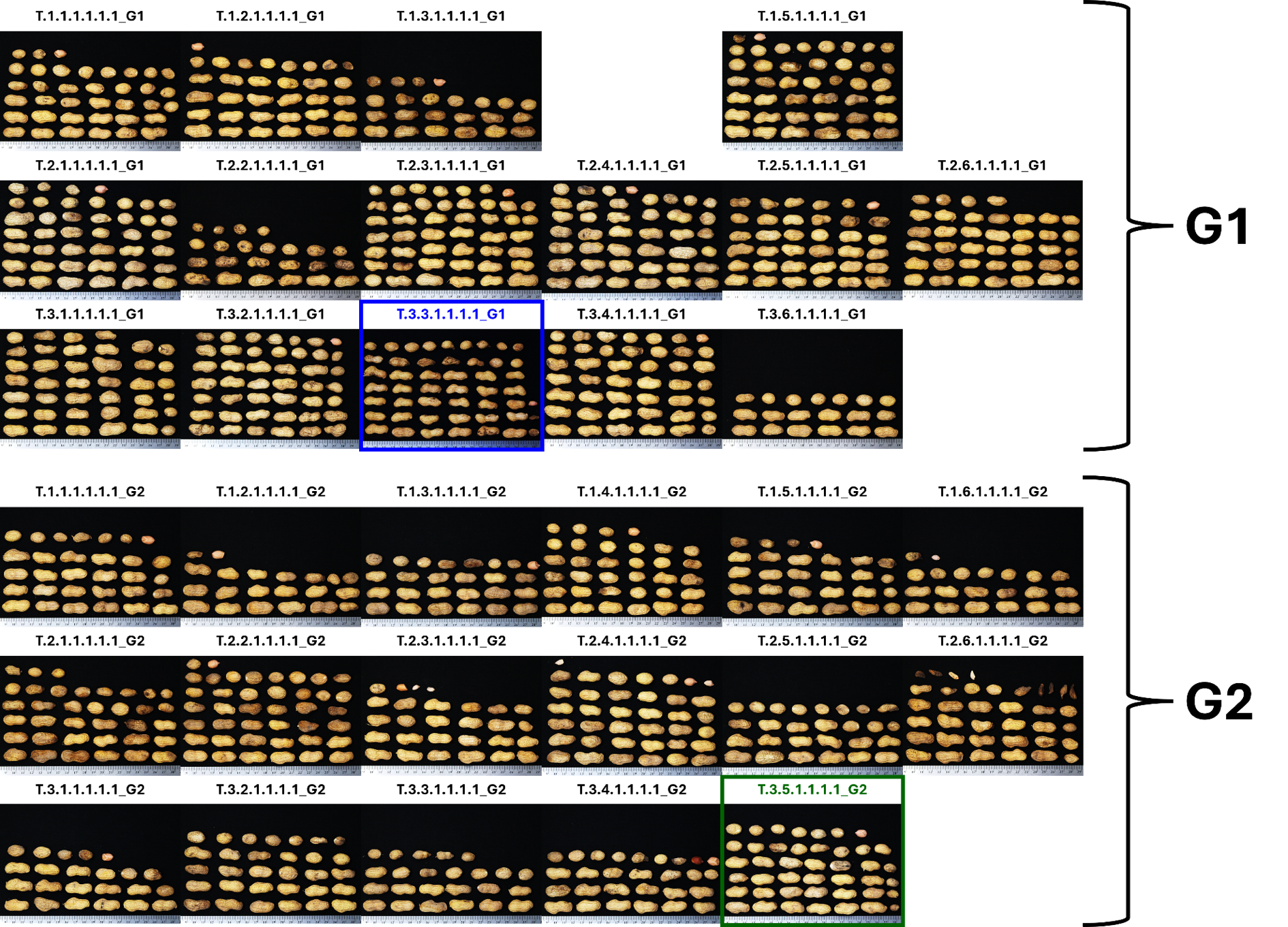
**

**Figure S8: Photographic images of generation 6 ‘Tifrunner’ plants cultivated in greenhouse 1 and 2.** T.3.3.1.1.1.1_G1, shown in blue, exhibits a B-subgenome deletion at the top of chromosome set 01 and reduced pod width and seed weight. T.3.5.1.1.1.1_G2, shown in green, displays an ABBB genomic composition at the top of chromosome set 05.

**
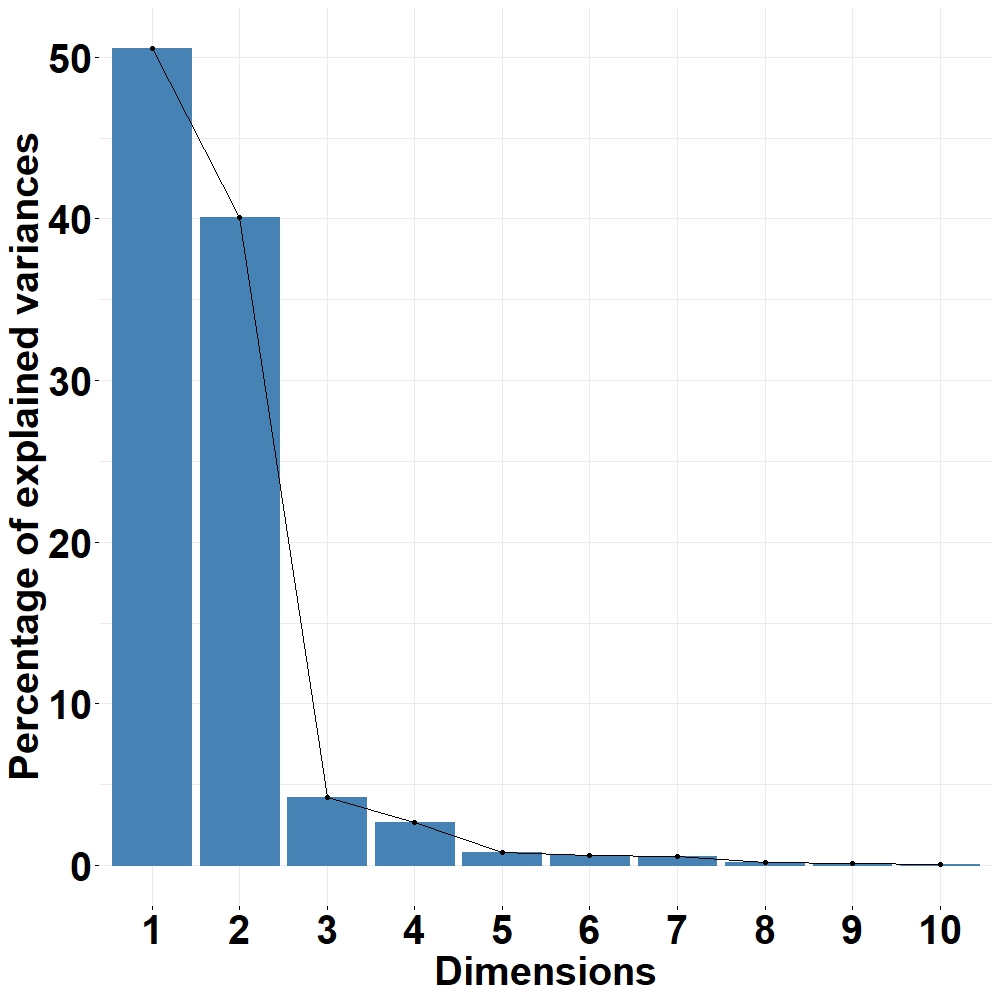
**

**Figure S9: Scree plot of principal component analysis for pod traits in generation 6 and 7 ‘Tifrunner’.** The first two principal components account for the majority of phenotypic variability, explaining 51% and 40%, respectively.

**
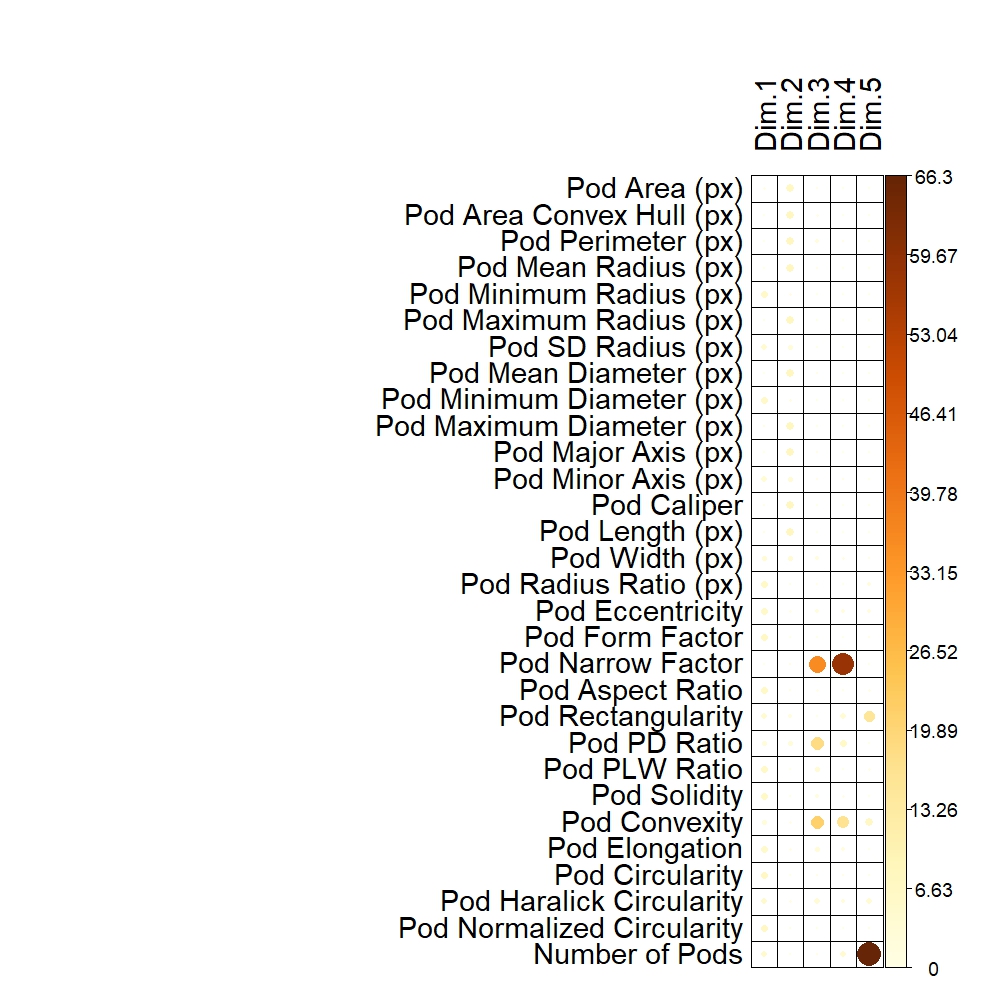
**

**Figure S10:** **Contribution of pod phenotypic variables to the first five dimensions of principal component analysis in ‘Tifrunner’ lineages.**

**
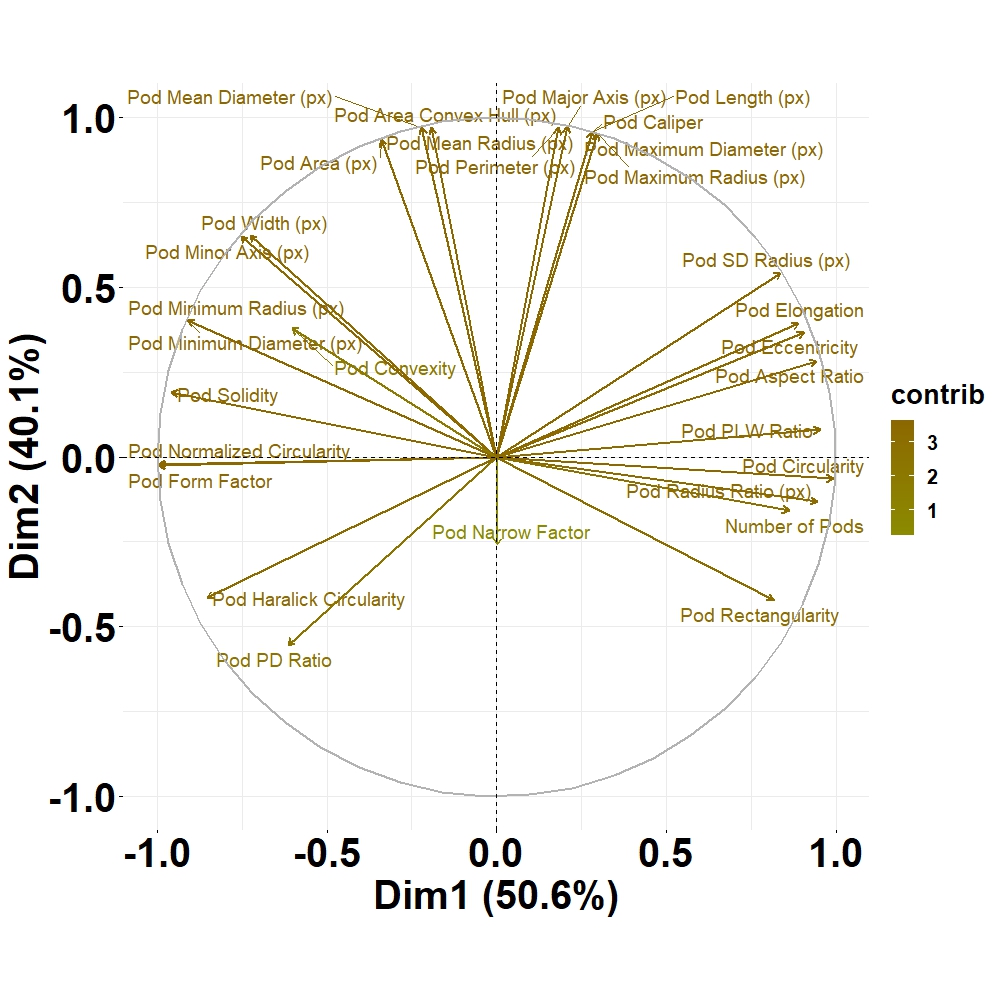
**

**Figure S11:** **Contribution and correlation of phenotypic traits in principal component analysis of ‘Tifrunner’ pod data.**

**
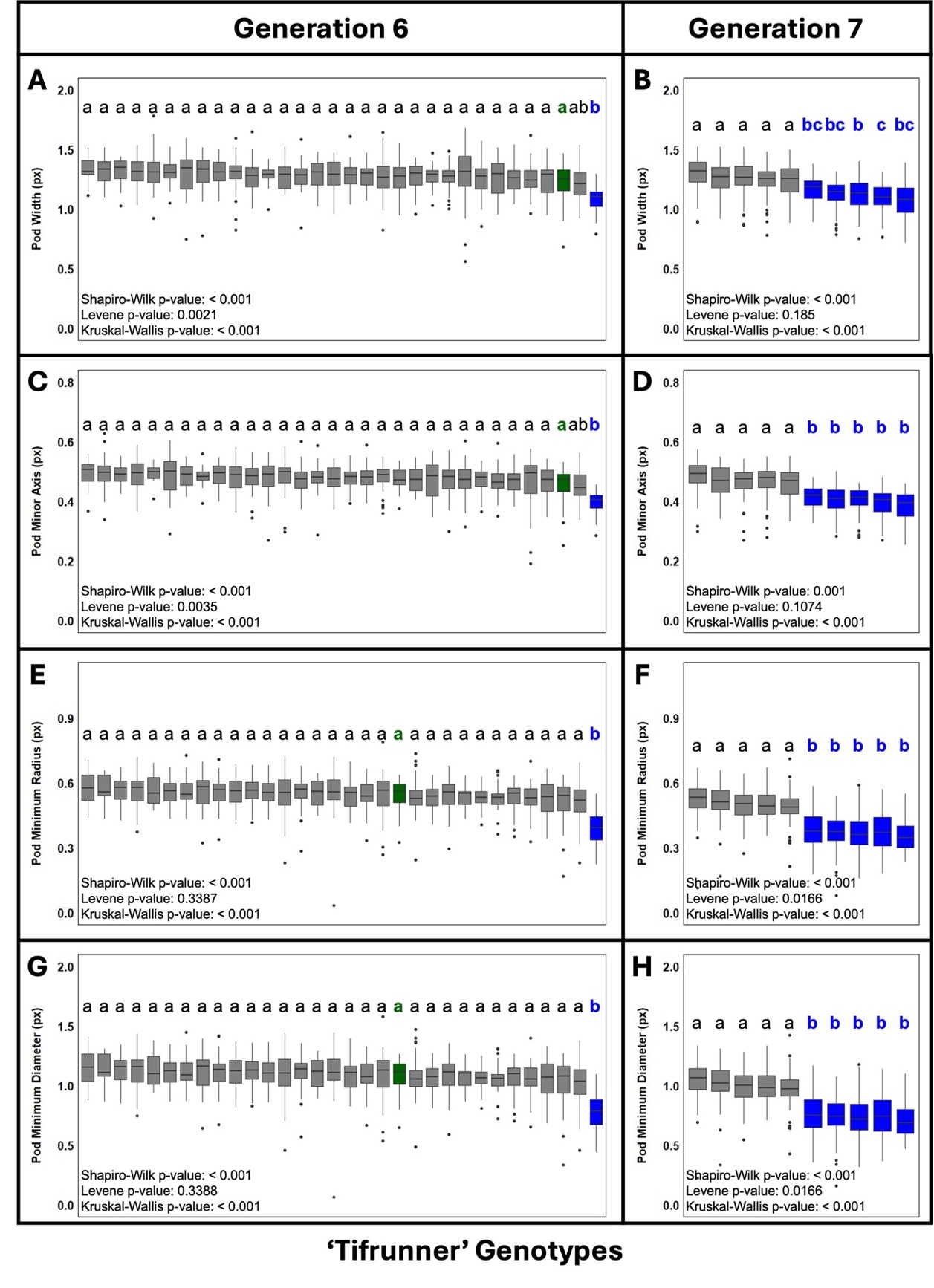
**

**Figure S12:** **‘Tifrunner’ lineage with a deletion at the top of chromosome B01 shows significantly lower pod trait values.** Box plots show pod width (A, B), pod minor axis (C, D), pod minimum radius (E, F), and pod minimum diameter (G, H) for generations 6 and 7. Normality was assessed using the Shapiro-Wilk test, homoscedasticity with Levene's test, and group differences with the Kruskal-Wallis test. P-values for each test are provided. Distinct letters indicate statistically significant differences among ‘Tifrunner’ plants based on Dunn’s test. Blue denotes lineage with a deletion on chromosome set 01, green represents plant with an ABBB genome composition on chromosome set 05, and grey indicates lineages without large-scale genetic instability events.
